# Combined oral and topical antimicrobial therapy for male partners of women with bacterial vaginosis: Acceptability, tolerability and impact on the genital microbiota of couples - A pilot study

**DOI:** 10.1371/journal.pone.0190199

**Published:** 2018-01-02

**Authors:** Erica L. Plummer, Lenka A. Vodstrcil, Jennifer A. Danielewski, Gerald L. Murray, Christopher K. Fairley, Suzanne M. Garland, Jane S. Hocking, Sepehr N. Tabrizi, Catriona S. Bradshaw

**Affiliations:** 1 Department of Molecular Microbiology, Murdoch Children’s Research Institute, Melbourne, Victoria, Australia; 2 Department of Microbiology and Infectious Diseases, The Royal Women’s Hospital, Melbourne, Victoria, Australia; 3 Melbourne Sexual Health Centre, Alfred Health, Melbourne, Victoria, Australia; 4 Central Clinical School, Faculty of Medicine, Nursing and Health Sciences, Monash University, Melbourne, Victoria, Australia; 5 Centre for Epidemiology and Biostatistics, Melbourne School of Population and Global Health, University of Melbourne, Parkville, Victoria, Australia; 6 Department of Obstetrics and Gynaecology, University of Melbourne, Melbourne, Victoria, Australia; 7 Department of Microbiology, The Royal Children’s Hospital, Melbourne, Victoria, Australia; Rush University, UNITED STATES

## Abstract

**Objectives:**

Recurrence following recommended treatment for bacterial vaginosis is unacceptably high. While the pathogenesis of recurrence is not well understood, recent evidence indicates re-infection from sexual partners is likely to play a role. The aim of this study was to assess the acceptability and tolerability of topical and oral antimicrobial therapy in male partners of women with bacterial vaginosis (BV), and to investigate the impact of dual-partner treatment on the vaginal and penile microbiota.

**Methods:**

Women with symptomatic BV (Nugent Score of 4–10 and ≥3 Amsel criteria) and their regular male sexual partner were recruited from Melbourne Sexual Health Centre, Melbourne, Australia. Women received oral metronidazole 400mg twice daily (or intra-vaginal 2% clindamycin cream, if contraindicated) for 7-days. Male partners received oral metronidazole 400mg twice daily and 2% clindamycin cream topically to the penile skin twice daily for 7-days. Couples provided self-collected genital specimens and completed questionnaires at enrolment and then weekly for 4-weeks. Genital microbiota composition was determined by 16S rRNA gene sequencing. Changes in genital microbiota composition were assessed by Bray-Curtis index. Bacterial diversity was measured by the Shannon Diversity Index.

**Results:**

Twenty-two couples were recruited. Sixteen couples (76%) completed all study procedures. Adherence was high; most participants took >90% of prescribed medication. Medication, and particularly topical clindamycin in males, was well tolerated. Dual-partner treatment had an immediate and sustained effect on the composition of vaginal microbiota (median Bray-Curtis score day 0 versus day 8 = 0.03 [IQR 0–0.15], day 0 vs day 28 = 0.03 [0.02–0.11]). We observed a reduction in bacterial diversity of the vaginal microbiota and a decrease in the prevalence and abundance of BV-associated bacteria following treatment. Treatment had an immediate effect on the composition of the cutaneous penile microbiota (median Bray-Curtis score day 0 vs day 8 = 0.09 [0.04–0.17]), however this was not as pronounced at day 28 (median Bray-Curtis score day 0 vs day 28 = 0.38 [0.11–0.59]). A decrease in the prevalence and abundance of BV-associated bacteria in the cutaneous penile microbiota was observed immediately following treatment at day 8.

**Conclusion:**

Combined oral and topical treatment of male partners of women with BV is acceptable and well tolerated. The combined acceptability and microbiological data presented in this paper supports the need for larger studies with longer follow up to characterize the sustained effect of dual partner treatment on the genital microbiota of couples and assess the impact on BV recurrence.

## Introduction

Bacterial vaginosis (BV) is a common condition and is associated with adverse pregnancy outcomes, increased risk of pelvic inflammatory disease, and increased susceptibility to HIV and other sexually transmitted infections (STIs)[1–4]. It is a dysbiosis characterized by a decrease in the abundance of *Lactobacillus* spp. and an increase in the number and diversity of anaerobic bacteria, collectively known as BV-associated bacteria[5, 6]. BV-associated bacteria identified to date include *Gardnerella vaginalis*, *Prevotella* spp., *Sneathia* spp., *Atopobium vaginae*, *Megasphaera* spp., Clostridia-like bacteria (known as BVAB-1, BVAB-2 and BVAB-3) and others[5, 7–10].

Current treatments are associated with unacceptably high recurrence (>50% within 6–12 months)[11, 12]. Possible mechanisms for recurrence include reinfection from a sexual partner or endogenous source, persistence of BV-associated bacteria following treatment and failure to recolonize with desirable *Lactobacillus* spp.[13, 14]. There is strong observational evidence that sexual activity plays a key role in both BV acquisition and recurrence[15–18]. Meta-analysis has shown associations between lack of condom use and exposure to new or multiple sexual partners with BV [19]. Additionally, two cohort studies of women who have sex with women reported a significant association between acquiring BV and reporting a new partner or a partner with BV[18, 20].

Microbiological data support the contribution of sexual transmission to the pathogenesis of BV through the exchange of BV-associated bacteria between sexual partners. The coronal sulcus and distal urethra can harbour BV-associated bacteria[21], and male partners of women with BV are reported to have an increased abundance of BV-associated bacteria in their penile skin and urethral microbiota compared to male partners of women without BV[22, 23].

Despite the strong evidence for sexual transmission of BV, randomised controlled trials (RCTs) of male partner-treatment have failed to reduce BV recurrence[24–29]. A recent Cochrane review rated the quality of the evidence as low to very low[30], and the discrepancy between current epidemiological evidence and the results of past RCTs is likely due to methodological limitations[31]. In addition none evaluated topical antibiotic therapy for males. We hypothesise that while urethral organisms are more likely to be effectively targeted by oral antibiotics, cutaneous colonisers of the coronal sulcus are more likely to be cleared by topical antibiotics. Thus it is plausible that combined oral and topical antimicrobial therapy is required to effectively clear BV-associated bacteria from both the coronal sulcus and distal urethra. Topical therapy is also likely to be particularly important in uncircumcised males who have a high abundance of sub-preputial BV-associated bacteria[22]. Male circumcision has been shown to reduce detection of BV-associated genera in males[32] and to reduce the risk of BV acquisition in women[33, 34], providing further evidence that cutaneous carriage of BV-associated bacteria plays an important role in the pathogenesis of BV acquisition and recurrence.

The primary objective of this pilot study was to assess the acceptability and tolerability of combined topical and oral antimicrobial therapy in male partners of women with BV. Our secondary objective was to investigate the impact of dual-partner treatment (i.e. treatment of both the female with BV and her male partner) on the genital microbiota of couples. There are no published data on the effect of antimicrobials used for BV treatment on the penile skin and urethral microbiota. Tolerability and microbiota data are needed to provide an evidence base to inform larger clinical trials of combined topical and oral therapy in males.

## Methods

### Participants, recruitment and intervention

Recruitment for this study was conducted from August 2015 to February 2016 at Melbourne Sexual Health Centre (MSHC), Australia, and the sample size was determined by funds available for this pilot. Women presenting with vaginal symptoms were routinely tested for BV by the Nugent and Amsel methods. BV was defined as a Nugent score [NS] of 4–10 and ≥ 3 Amsel criteria and was treated with oral metronidazole 400 mg twice daily for seven days or 2% vaginal clindamycin cream as one applicator vaginally for seven nights if metronidazole was contraindicated or declined. Women diagnosed and treated for BV who had a regular male partner and expressed interest in the study were referred to a research nurse who screened them for eligibility. Women were eligible if they were 18 to 55 years old, were being treated for symptomatic BV and had a single regular male sexual partner who was willing to be enrolled in the trial (women were asked to confirm if it was likely that their male partner would agree participate). A regular partner was defined for the purpose of this study as someone who was considered by the female to be a boyfriend or partner. Women were ineligible if they were HIV positive, pregnant or breast feeding, diagnosed with current PID, if they were allergic to study medication, or had other concurrent sexual partners.

Male partners of eligible women were recruited either in clinic following onsite consultation, or during a phone consultation with a clinician and research nurse; an electronic medical record for these male participants was created. Males were ineligible if they were: HIV positive, allergic to metronidazole and/or clindamycin, or had other concurrent sexual partners.

Males received oral metronidazole 400mg twice daily and were instructed to apply a 2 cm diameter volume of 2% clindamycin cream topically to the head of the penis and upper shaft (under the foreskin if uncircumcised) twice daily for seven days. Where possible, the male partner started treatment on the same day as his female partner; however, treatment could be commenced within a week of the female commencing therapy.

Participants received a voucher as reimbursement for their time (valued up to a maximum of AUD $50 dependent on number of study visits completed).

### Study procedures

Before commencing treatment, women completed a questionnaire recording demographic, behavioural, clinical and contraceptive information. Women provided two self-collected high-vaginal swabs (using Copan flocked swabs) for Nugent scoring and microbiota analysis. Males completed a questionnaire recording demographic and behavioural information, and provided a self-collected penile swab and a urine swab for microbiota analysis. The penile swab was obtained by rubbing a Copan flocked swab moistened with sterile water around the coronal sulcus and over the glans of the penis. Males were instructed to rub the swab firmly twice around the coronal sulcus before using the same swab to rub the glans of the penis. If the male was uncircumcised he was instructed to pull pack his foreskin before collecting the swab. For the urine swab, males urinated the first 20 mL of urine into a urine pot and dipped a Copan flocked swab into the collected urine to facilitate return of specimens by post.

Participants returned weekly questionnaires and self-collected genital specimens for four weeks following completion of treatment (8, 14, 21 and 28 days post treatment). At each time point females provided a vaginal swab and a vaginal smear for Nugent scoring; males provided a penile swab and a urine swab. Questionnaires and specimens were returned by mail. Participants were asked to either abstain from penile-vaginal sex or to have protected sex during the treatment period (days 0 to 7).

### Outcomes

#### Primary outcome

The primary outcome was to assess male participant acceptability and tolerability of treatment. Adherence and side effects to treatment were self-reported on day 8 at the end of the treatment period, providing a measure of acceptability of the trial. Couples were included in the analysis of the primary outcome if both the male and female partner completed the day 8 questionnaire.

#### Secondary outcome

The secondary outcome was the impact of dual-partner treatment on the genital microbiota of couples, assessed at baseline (i.e. day 0), day 8 and 28.

### Laboratory methods

#### Nugent scoring and specimen storage

Although this study was not powered to measure BV recurrence, vaginal smears underwent blinded Nugent Scoring[35] by an experienced microscopist so that we could record whether or not BV recurred within the 28 day follow-up period. All swabs were rotated in 1ml RNAlater (Life Technologies; Thermo Fisher Scientific, Waltham, USA) and stored at -80°C for microbiota analysis.

#### DNA extraction, bacterial load quantification and sequencing

DNA was extracted from 200 μL of specimens on an automated MagNA Pure 96 isolation and purification system using the DNA and Viral NA small volume kit (Roche Diagnostics, Mannheim, Germany) according to the manufacturer’s protocol. DNA was eluted in a final volume of 100 μL, followed by a quantitative β globin assay to assess specimen adequacy, as previously described[36]. A quantitative 16S PCR was performed to report total bacterial load (16S rRNA gene copies per 5 μl of extracted DNA) using the broad range primer pair fD1 mod and 16S1RR-B, with 515F modified as a Taqman probe[37]. Specimens with insufficient DNA for amplification were re-extracted using an alternate methodology (S1 File). Twenty negative control samples were included to facilitate identification of reagent contaminants (S1 Table). Dual indexed universal primers Bakt_341F (CCTACGGGNGGCWGCAG) and Bakt_805R (GACTACHVGGGTATCTAATCC)[38, 39] were used for PCR amplification of the V3-V4 hypervariable regions of the 16S rRNA gene, as previously described[40]. Specimens and controls were sequenced on the Illumina MiSeq platform (Micromon, Monash University, Victoria, Australia).

### Sequence analysis

Forward and reverse reads were paired using the Paired-End reAd mergeR (PEAR) v0.9.6[41]. Data were demultiplexed using QIIME (version 1.8.0)[42] and reads with a quality score less than 20 were discarded. Tagcleaner (standalone v0.16)[43] was used to trim primers and heterogeneity spacers from reads. Chimeras were filtered using the reference mode of UCHIME (as integrated in USEARCH v8.0 1517)[44] using the 16S rRNA Gold reference database[45].

Open reference operational taxonomic unit (OTU) picking was performed in QIIME using the UCLUST algorithm[46] at 97% identity. Taxonomy of OTUs was assigned at 97% similarity using the UCLUST consensus taxonomy assigner and SILVA reference database (v111)[47]. Species level information was obtained for reads assigned to the *Lactobacillus* genus using a BLAST[48] search of the 16S ribosomal RNA BLAST database. Species taxonomic information was used where the percent identity for the top BLAST hit was greater than 97%.

Initially, unassigned reads and OTUs with less than three sequences were discarded. The OTU table was then screened for contaminants. OTUs were flagged as contaminants and filtered from the OTU table if they were present in all control specimens or previously reported as common sequencing contaminants and were not expected in the clinical context. Similar approaches have been discussed previously[49, 50](S2 Table). A total of 6,674,016 reads remained after post-processing and contaminant removal.

Specimens were rarefied to an even sampling depth (1,100 reads) prior to analysis. Two cutaneous penile and thirteen urine specimens did not produce an adequate number of reads and were excluded from further analysis. As a result, there were insufficient urine specimens to enable paired comparisons before and after treatment for participants and between couples.

Sequencing reads are available in NCBI Short Read Archive (SRA, http://www.ncbi.nlm.nih.gov/sra) under BioProject ID PRJNA398590.

### Visualisation of the genital microbiota

Using R Studio [V0.98.1103, Boston, USA] employing R3.2.0[51], heatmaps and associated dendrograms were generated using the vegan and gplots packages [52, 53] and were based on hierarchical clustering using the Bray-Curtis index. The 30 most abundant bacterial taxa for each specimen type were included in the heatmap analysis.

### Statistical methods

Statistical analyses were performed using Stata/IC (Version 14, StataCorp LP, College 167 Station, USA). The proportion of females and males who were retained in the study and who adhered to medication was calculated. Adherence was calculated as number of tablets taken or doses applied as a proportion of the total number of tablets or doses prescribed. Comparisons of log-transformed bacterial loads between specimen types were made using Welch’s t-test, and between paired specimens using the paired t-test.

Bray-Curtis scores were calculated using the vegan package between paired specimens from each participant to investigate the immediate (day 0 and 8) and sustained (day 0 and 28) effect of treatment on the composition of the vagina and cutaneous penile microbiota. Scores were given a value from zero (substantial change in the presence or abundance of bacterial taxa) to one (minimal change). Alpha diversity was expressed as the effective number of taxa (i.e. the exponent of the Shannon Diversity Index) using the Picante package for R[54]. Changes in alpha diversity were assessed by the Wilcoxon signed-rank test.

Prevalence of each taxon was calculated as the number of specimens positive for a specific taxon at time point *A* for specimen type *X* as a proportion of the total number of specimens available at time point *A* for specimen type *X*. Abundance of each taxon was calculated as the number of sequences for a specific taxon in specimen *A* as a proportion of the total number of sequences in specimen *A*; abundance of taxa was summarised by specimen type and time point using descriptive statistics (mean, median, range and interquartile range [IQR]). Changes in the prevalence and abundance of specific bacterial taxa between pre and post treatment specimens were assessed by McNemar’s chi-squared test and the Wilcoxon signed-rank test, respectively. The 30 most abundant bacterial taxa for each specimen type were included in prevalence and abundance analyses.

We measured the impact of sexual partnerships on the genital microbiota of sexual partners by: 1) comparing the similarity of the genital microbiota of partners to non-partners, and 2) investigating the correlation of prevalent taxa in the vaginal and cutaneous penile microbiota of partners. We used the approach of Zozaya et al[23] to compare the similarity in bacterial communities of sexual partners to non-partners, with the following modifications: Bray-Curtis scores were used as the distance measure (as described above) and the Wilcoxon signed-rank test was used to measure statistical significance. Spearman’s rho was used to assess the correlation between prevalent taxa in the vaginal microbiota of women and the same taxa in the cutaneous penile microbiota of their sexual partner at three time points: baseline, day 8 and day 28. Bacterial taxa present in at least 30% of vaginal specimens collected at baseline, day 8 and day 28 were included in the correlation analysis.

A *p-value*<0.05 was deemed significant. *P-value* false discovery rate adjustment for multiple comparisons was performed where required using the Benjamini-Hochberg procedure; a *q*-value <0.05 was deemed significant.

### Ethics

This trial received ethics approval from the Human Research and Ethics Committee of the Alfred Hospital, Melbourne, Australia (Project number 264/15). Written informed consent was obtained from all participants.

In compliance with the requirements of the Alfred Hospital Human Ethics Committee, this study was prospectively filed with Australia’s Therapeutics Good Administration via the Clinical Trial Notification scheme (CTN; clinical trial: CT-2015-CTN-00884-1). It was retrospectively registered with the Australian New Zealand Clinical Trials Registry (ANZCTR; ACTRN12617001302347).

## Results

Results are published in accordance with the Transparent Reporting of Evaluations with Non-randomised Designs (TREND) statement (S2 File)[55].

### Participant recruitment, retention and baseline characteristics

**Fig 1:**
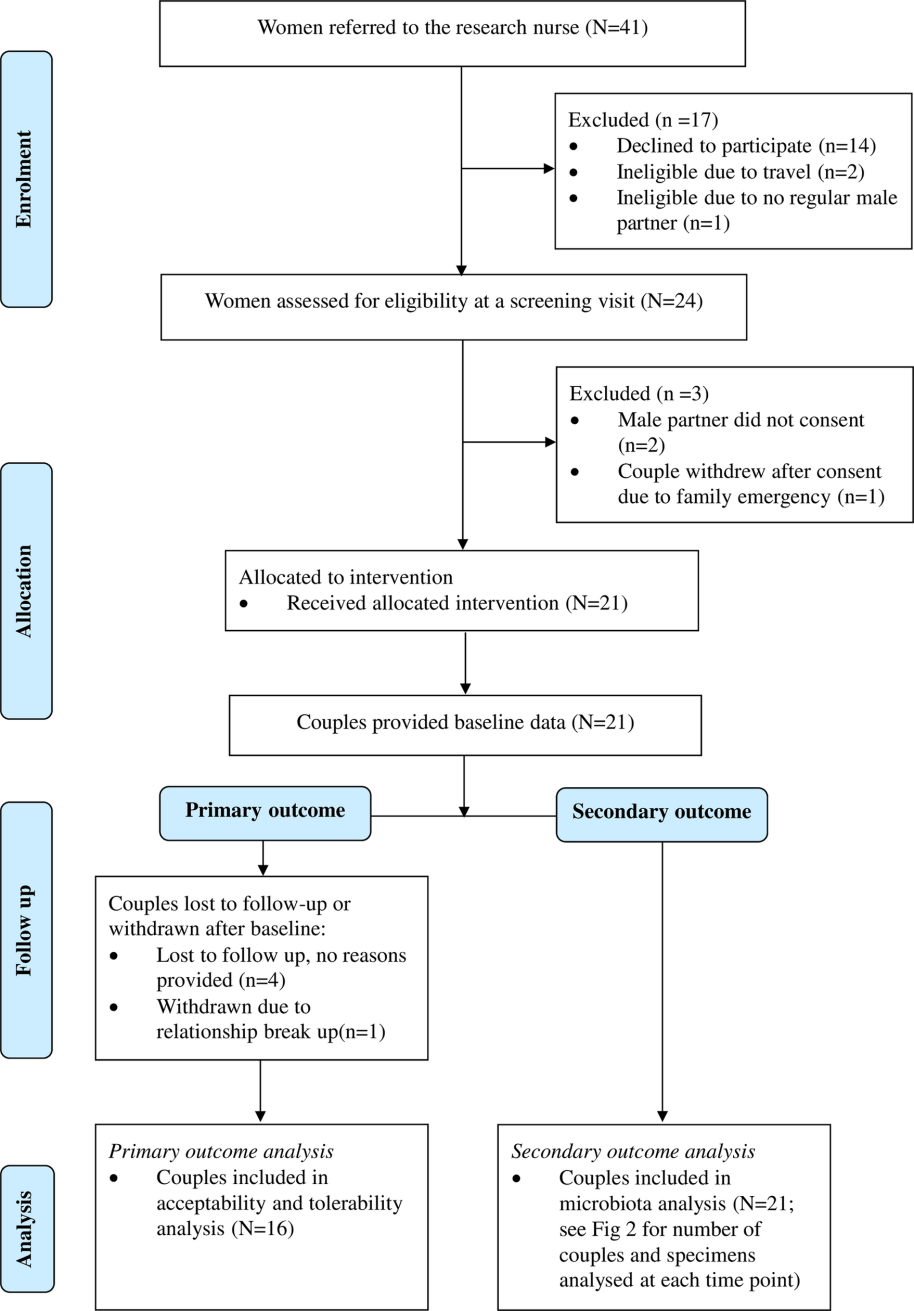
Participant flowchart. Participant flowchart detailing number of women screened for eligibility, resulting number of couples recruited to study and their progression through the study period. LTFU, lost to follow up.

Forty-one women were referred to the research nurse, 14 women declined and three were deemed ineligible. Twenty-four women were invited to attend a screening visit between the designated recruitment period from August 2015- February 2016 and of these, 22 male partners (92%; 22 couples) were co-enrolled. Two male partners declined participation after the female had been screened (Fig 1). Male partners were recruited either by phone and electronic record consultation (n = 14, 64%), or by on site clinic consultation (n = 8). Twenty-one couples received study medication and provided baseline data (95%) as one couple withdrew before completing any study procedures due to end of relationship. After providing baseline data, four couples were lost to follow-up (LTFU) (19%) and one withdrew due to a family emergency. Adherence, tolerability and follow-up data were available for 16 couples (76%).

The mean age at baseline was 28.6 years (SD, 6.4 years) for women and 33.1 years (SD, 9.1 years) for men (Table 1) for the 21 couples who received treatment. Approximately half of participants were Australian born (52 and 55%, respectively for females and males). Smoking was reported by eight women (38%) and nine men (45%). The median duration of relationship between couples was nine months (IQR, 3–12 months). All couples reported unprotected vaginal sex in the month prior to recruitment; seven couples (35%) reported unprotected anal sex during this period as well. A history of BV was reported by 17 women (81%), eleven women (52%) were receiving hormonal contraception and two (10%) performed vaginal douching. Four men (19%) were circumcised.

**Table 1:** Demographic and behavioural characteristics of couples at baseline.

Baseline (Day 0)		
	Female (N = 21)	Male (N = 21)
Mean age at baseline, years (SD)	28.6 (6.4)	33.1 (9.1)
Country of Birth		
Australia	11 (52)	11 (55) ^a^
Other	10 (48)	9 (45)
Any smoking		
No	13 (62)	11(55) ^a^
Yes	8 (38)	9 (45)
Past history of BV		
No	4 (19)	-
Yes	17 (81)	-
Mean months since last BV episode (SD)	8.3 (11.7)	-
Any hormonal contraception		
No	10 (48)	-
Yes	11 (52)	-
Any douching		
No	18 (90) ^a^	-
Yes	2 (10) ^a^	-
Circumcised		
No	-	17 (81)
Yes	-	4 (19)
Number of sexual partners in last 12 months^b^		
<4	10 (50) ^a^	12 (57)
≥4	10 (50)	9 (43)
Median duration of partnership, months (IQR)	9 (3–12) ^a^	9 (3–12)
Median time since last penile-vaginal sex with other partner, months (IQR)^c^	3 (2–10)	4 (1–8)
Mean # of oral sex acts received per month (SD)	11.9 (10.8) ^a^	-
Mean # of vaginal sex acts per month (SD)^d^	21.3 (12.1) ^a^	16.9 (11.6) ^e^
Any unprotected vaginal sex in last month		
No	0 ^a^	0 ^a^
Yes	20 (100)	20(100)
Any unprotected anal sex in last month		
No/ not practiced	13 (65) ^a^	13 (68) ^e^
Yes	7 (35)	6 (32)
Antibiotics taken in last month		
No	11 (55) ^a^	19 (90)
Yes	9 (45) ^f^^,^^g^	2 (10) ^f^
Vaginal treatments used in last month		
No	18 (90) ^a^	-
Yes	2 (10)	-
Treatments on penis used in last month		
No	-	18 (90) ^a^
Yes	-	2 (10)

Data presented as n(%) unless otherwise specified; Abbreviations: SD, standard deviation; IQR, Interquartile range

^a^ Missing data (n = 1)

^b^ Includes both male and female sexual partners

^c^ If participants reported a sexual partner/s in the last 12 months other than their regular partner, they were asked to report the time since last penile-vaginal sex with the most recent other sexual partner. Data provided from nine women and six males.

^d^ Discrepancies are a result of independent reporting by the female and her male partner.

^e^ Missing data (n = 2)

^f^ Includes one couple treated for gonorrhoea at enrolment and one couple treated for chlamydia at enrolment

^g^ Four women reported receiving metronidazole in the month prior to enrolment. Other antibiotics reported were amoxicillin, trimethoprim, nitrofurantoin and gentamicin.

All women had ≥3 Amsel criteria and a Nugent Score of 4–10 at baseline; 19 (90%) had a Nugent score of 7–10 (Table 2). One couple was positive for chlamydia and was prescribed azithromycin (single 1g oral dose); one female was positive for gonorrhoea and she and her male partner were prescribed azithromycin (single 1g oral dose) and ceftriaxone (500mg intramuscular injection).

**Table 2:** Clinical and laboratory characteristics of females.

	Baseline (day 0) (N = 21)^a^	Study Endpoint (day 28) (N = 16)^a^
Self-reported symptoms		
Vaginal discharge		
No	3 (14)	11 (69)
Yes	18 (86)	5 (31)
Vaginal odour		
No	2 (10)	15 (94)
Yes	19 (90)	1 (6)
Mean time since LNMP, days (SD) ^b^	23 (18)	23 (24)
Nugent score		
0–3	0 (0)	12 (75)
4–6	2 (10)	3 (19)
7–10	19 (90)	1 (6)^c^

Data presented as n(%) unless otherwise specified; Abbreviations: LNMP, Last known menstrual period; SD, standard deviation.

^a^ Clinical and laboratory data is available for 21 women at baseline and 16 women at day 28 as one couple withdrew and four couples were lost to follow up after providing baseline data.

^b^ LNMP missing for two participants at baseline and one participant at study endpoint.

^c^ A second woman had a Nugent score of 8 at day 14. She was subsequently treated with vaginal clindamycin and had a Nugent score of 4 at day 28.

### Acceptability and tolerability

Of the 16 couples who provided adherence and tolerability data, 14 women received oral metronidazole and two requested vaginal clindamycin; all males received both oral metronidazole and topical clindamycin. For 15 of the 16 couples, the male and female partner started treatment within four days of each other (10 started simultaneously), and for one couple, the male partner started treatment a week following the female. Self-reported adherence to metronidazole was high; 13 females (93%) and 14 males (88%) took over 90% of tablets (Table 3). Self-reported adherence was lower with clindamycin; eleven males (69%) applied over 90% of clindamycin doses and of the two females who received vaginal clindamycin, one applied all doses but the other missed one application.

**Table 3:** Treatment adherence and side effects.

	Female (N = 16)	Male (N = 16)
Prescribed Metronidazole (oral)^a^	14 (87.5)	16 (100)
Self-reported adherence to metronidazole		
Percent of tablets taken		
100% tablets taken	13 (93)	11 (69)
>90% tablets taken	13 (93)	14 (88)
>70% tablets taken	14 (100)	16 (100)
Prescribed Clindamycin (topical)^a^	2 (12.5)	16 (100)
Self-reported adherence to clindamycin		
Percent of doses applied		
100% doses applied	1 (50)	9 (56)
>90% doses applied	1 (50)	11 (69)
>70% doses applied	2 (100)	15 (94)
>50% doses applied	2 (100)	16 (100)
Adverse effects^b^		
Nausea		
No	13 (81)	15 (94)
Yes	3 (19)	1 (6)
Vomiting		
No	16 (100)	16 (100)
Yes	0 (0)	0 (0)
Metallic taste		
No	13 (81)	15 (94)
Yes	3 (19)	1 (6)
Headache		
No	14 (87.5)	14 (87.5)
Yes	2 (12.5)	2 (12.5)
Vaginal irritation		
No	15 (94)	-
Yes	1 (6)	-
Irritation of penile skin		
No	-	16 (100)
Yes	-	0 (0)
Redness of penile skin		
No	-	15 (94)
Yes	-	1 (6)
Other		
No	11 (69)	13 (81)
Yes	5 (31) ^c^	3 (19)^d^

Data presented as n(%) unless otherwise specified

^a^ Oral metronidazole was standard treatment for females. Two females requested treatment with vaginal clindamycin.

^b^ No side effects reported for females treated with vaginal clindamycin

^c^ Other side effects: hungry all the time, got period when usually do not, tiredness, mild stomach pain, yeast infection.

^d^ Other side effects: dark urine, mild generalised body rash (itchy spots on upper torso and arms), upset stomach

Study medications were well tolerated by participants. The most commonly reported adverse effects were nausea and metallic taste for females (n = 3, 19%) and headaches for both males and females (n = 2, 12.5%; Table 3). One male experienced a mild body rash involving trunk and limbs (not involving the penis) on day six of treatment and was advised not to take the final day of study medication. The women who received clindamycin reported no adverse effects.

### Behavioural practices from baseline to day 28

During the treatment period (day 0 to 7) six of 16 couples reported unprotected vaginal sex, one couple reported unprotected anal sex and five couples reported oral sex (Table 4). All 16 couples reported unprotected vaginal sex between day 8 and 28, with one couple reporting unprotected anal sex during this time. One male ceased smoking and one male commenced smoking during the treatment period. One woman reported using condoms more frequently at day 28 and one woman reported a change from monthly to daily douching.

**Table 4:** Behavioural characteristics of couples during study period.

	Treatment Period (day 0 to 7)		Follow up period (day 8 to 28)	
	Female (N = 16)	Male (N = 16)	Female (N = 16)	Male (N = 16)
Any vaginal sex				
No	6 (37.5)	7 (44)	0 (0)	0 (0)
Yes	10 (62.5)	9 (56)	16 (100)	16 (100)
Any unprotected vaginal sex				
No	11 (69)	11 (69)	0 (0)	0 (0)
Yes	5 (31)^a^	5 (31)^a^	16 (100)	16 (100)
Mean # of vaginal sex acts (SD)^b^	2.3 (4.8)	1.4 (1.5)	9.8 (6.6)	8.7 (5.9)
Any oral sex received				
No	11 (69)	-	2 (12.5)	-
Yes	5 (31)	-	14 (87.5)	-
Any anal sex^b^				
No	15 (94)	15 (94)	15 (94)	14 (87.5)
Yes	1 (6)	1 (6)	1 (6)	2 (12.5)
Any unprotected anal sex				
No	15 (94)	15 (94)	15 (94)	15 (94)
Yes	1 (6)	1 (6)	1 (6)	1 (6)
Exposure to new sexual partner reported				
No	16 (100)	16 (100)	16 (100)	15 (94)
Yes	0 (0)	0 (0)	0 (0)	1 (6)
Concomitant treatments				
Antibiotic	3 (19) ^c^^,^ ^d^	1 (6)^d^	1 (6) ^e^	0(0)
Antifungal	0 (0)	0(0)	6 (31) ^f^	2 (13) ^f^
Other	0 (0)	0 (0)	1(6) ^g^	1 (6) ^g^

Data presented as n(%) unless otherwise specified; Abbreviations: SD, standard deviation.

^a^ Five females and five males independently reported unprotected vaginal sex during the treatment period, representing a total of six couples who had unprotected sex during the treatment period.

^b^ Discrepancies are a result of independent reporting by the female and her male partner.

^c^ During treatment period, one female reported treatment with trimethoprim for a urinary tract infection and one reported receiving antibiotic treatment for a chest infection (did not specify treatment name)

^d^ This includes the couple who received treatment for gonorrhoea at enrolment.

^**e**^ One female was prescribed clindamycin between days 14 and 21 of study for BV recurrence.

^f^ Six women reported receiving treatment for thrush. One male reported receiving clotrimazole and one male reported ketoconazole

^g^ One female reported using other vaginal treatments for itchiness but did not provide details; one male reported using Vaseline® on the penile skin for dryness.

### Genital microbiota at baseline

**Fig 2:**
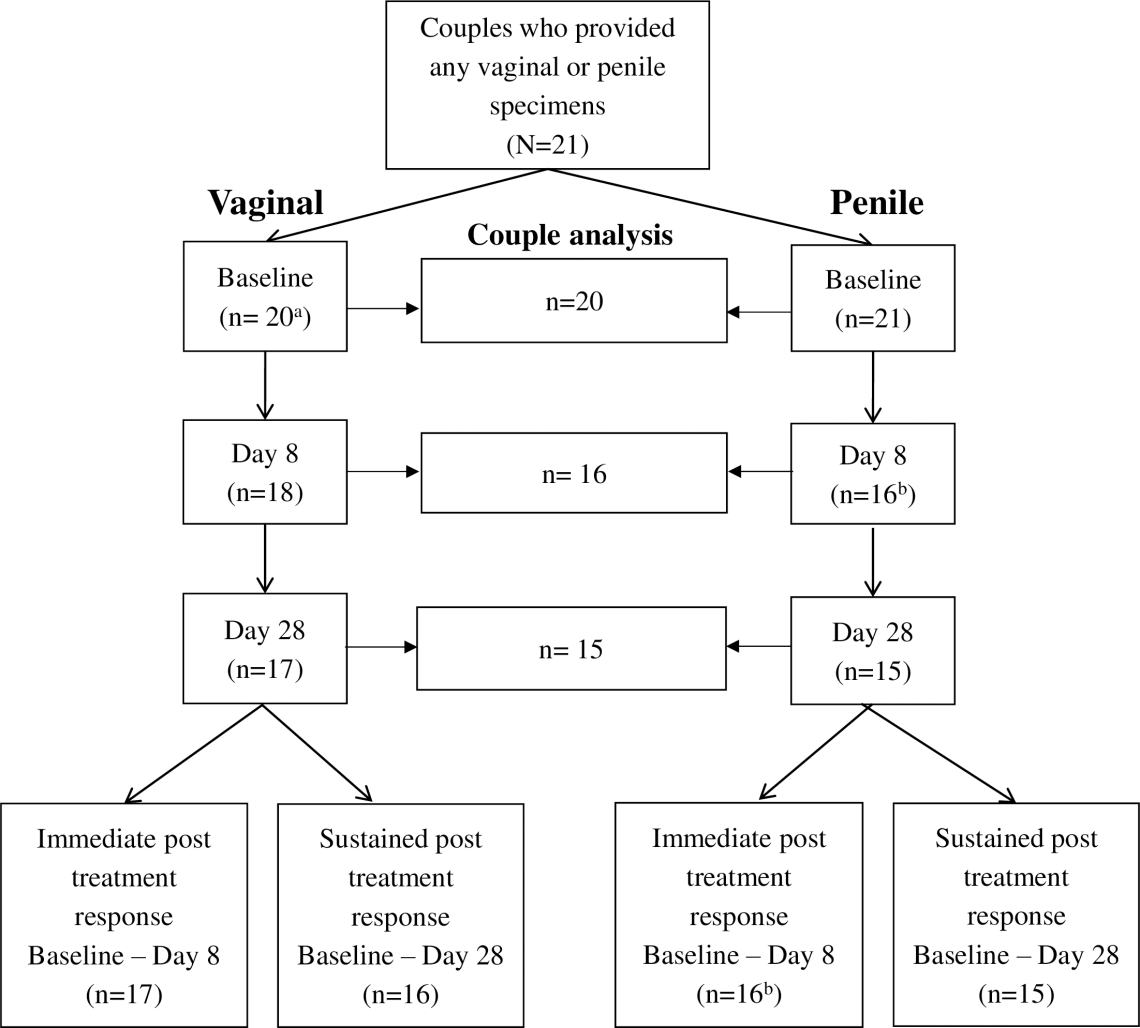
Specimen flowchart. Specimen flowchart detailing number of vaginal and penile skin specimens available for microbiota analysis at baseline, day 8 and day 28. Seventeen women provided vaginal specimens for day 0 and 8 paired comparisons, and 16 provided vaginal specimens for day 0 and 28 paired comparisons. Sixteen males provided cutaneous penile specimens for day 0 and 8 paired comparisons and 15 males provided cutaneous penile specimens for day 0 and 28 paired comparisons. The number of couples providing specimens at each time-point is also shown. ^a^baseline specimen was not available for one female; ^b^two d8 penile skin specimens failed to meet the sequence depth threshold and were substituted with day 14 specimens.

**Fig 3:**
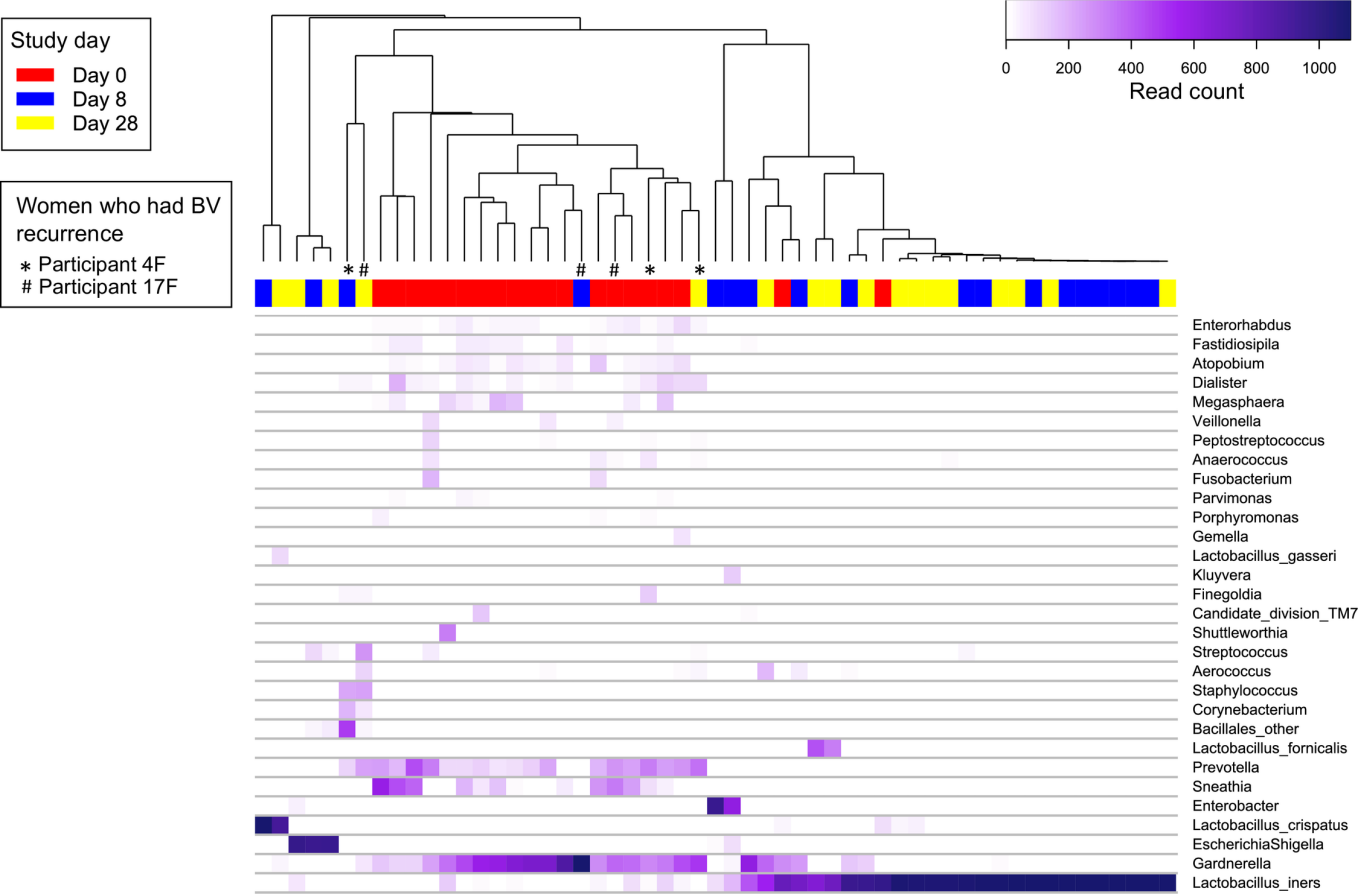
Heatmap of bacterial abundance from vaginal specimens collected at baseline, day 8 and day 28. Each vertical line represents the bacterial composition of one vaginal specimen. Only the 30 most abundant taxa found in vaginal specimens are included in the heatmap. Study day is displayed above the heatmap in red (day 0), blue (day 8) and yellow (day 28). Specimens collected from females who experienced BV recurrence during the study are indicated by * and # below the dendrogram.

**Fig 4:**
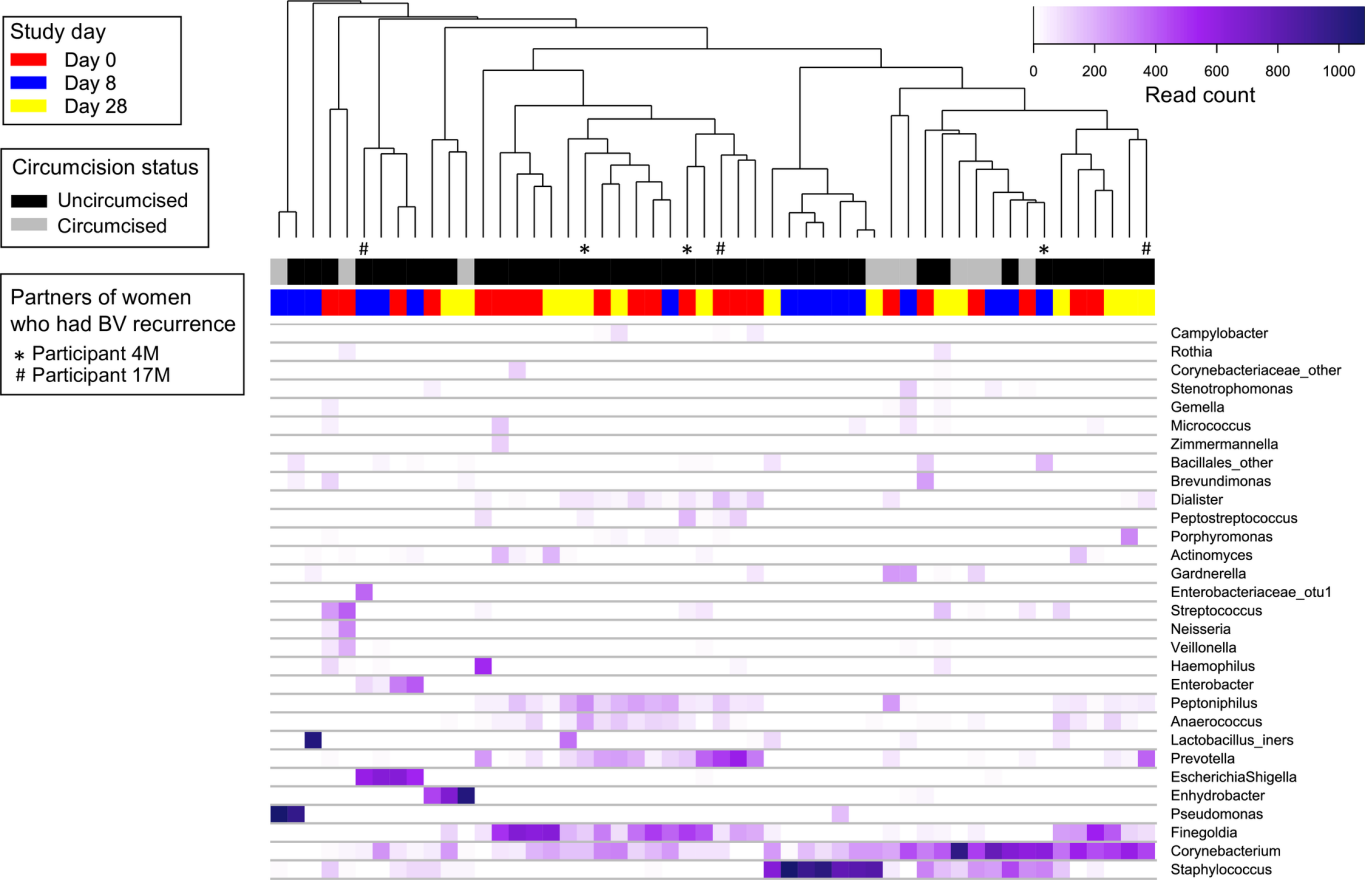
Heatmap of bacterial abundance from penile skin specimens collected at baseline, day 8 and day 28. Each vertical line represents the bacterial composition of one penile specimen. Only the 30 most abundant taxa found in penile specimens are included in the heatmap. Study day is displayed above the heatmap in red (day 0), blue (day 8) and yellow (day 28); circumcision status is displayed in black (uncircumcised) and grey (circumcised). Specimens collected from male partners of women who experienced BV recurrence during the study are indicated by * and # below the dendrogram.

Of the 21 couples who provided baseline data, there were 20 vaginal and 21 cutaneous penile baseline specimens available for analysis (Fig 2). BV-associated bacteria (specifically *Gardnerella*, *Prevotella* and *Sneathia*) were highly prevalent and abundant in baseline vaginal specimens (Fig 3). Two women had a high abundance of *Lactobacillus iners*. *Corynebacterium* and BV-associated bacteria (specifically *Finegoldia* and *Peptoniphilus*) were highly prevalent and abundant in in baseline cutaneous penile specimens (Fig 4).

Four baseline urine specimens were excluded as they did not meet the sequence depth threshold. Of the eight baseline urine specimens available for analysis, *Streptococcus* and *Corynebacterium* were the most prevalent taxa, though both were detected at low abundance (detected in seven of eight specimens; S1 Fig). Importantly, *Gardnerella* was detected at between 7–52% abundance in 5 of the 8 baseline urine specimens.

### Effect of dual partner treatment on the vaginal and penile microbiota

Using paired comparisons, we investigated the immediate post treatment response (day 0 and 8) and sustained post treatment response (day 0 and 28) effect of dual partner treatment on the overall composition and diversity of the genital microbiota, as well as the impact of treatment on the prevalence and abundance of key bacterial taxa present in the vagina and penile skin.

Seventeen women provided vaginal specimens for day 0 and 8 paired comparisons, and 16 provided vaginal specimens for day 0 and 28 paired comparisons. Sixteen males provided cutaneous penile specimens for day 0 and 8 paired comparisons and 15 males provided cutaneous penile specimens for day 0 and 28 paired comparisons. Two day 8 cutaneous penile specimens failed to meet the sequence depth threshold and were substituted with day 14 specimens (Fig 2).

Seven day 8 and five day 28 urine specimens were available for urethral microbiota analysis. Adequate paired urethral data (i.e. a baseline urine data and day 8 and/or day 28 urine data) was available for only two participants. As a result, we were not able to assess the effect of dual partner treatment on the urethral microbiota. For urine data that was able to be assessed, *Staphylococcus* and *Corynebacterium* were the most prevalent taxa at day 8 (detected in three specimens of seven); *L*. *iners* was the most prevalent taxa in urines at day 28 (detected in three of five specimens; S1 Fig).

#### Immediate and sustained effect of treatment on the vaginal microbiota

**Fig 5:**
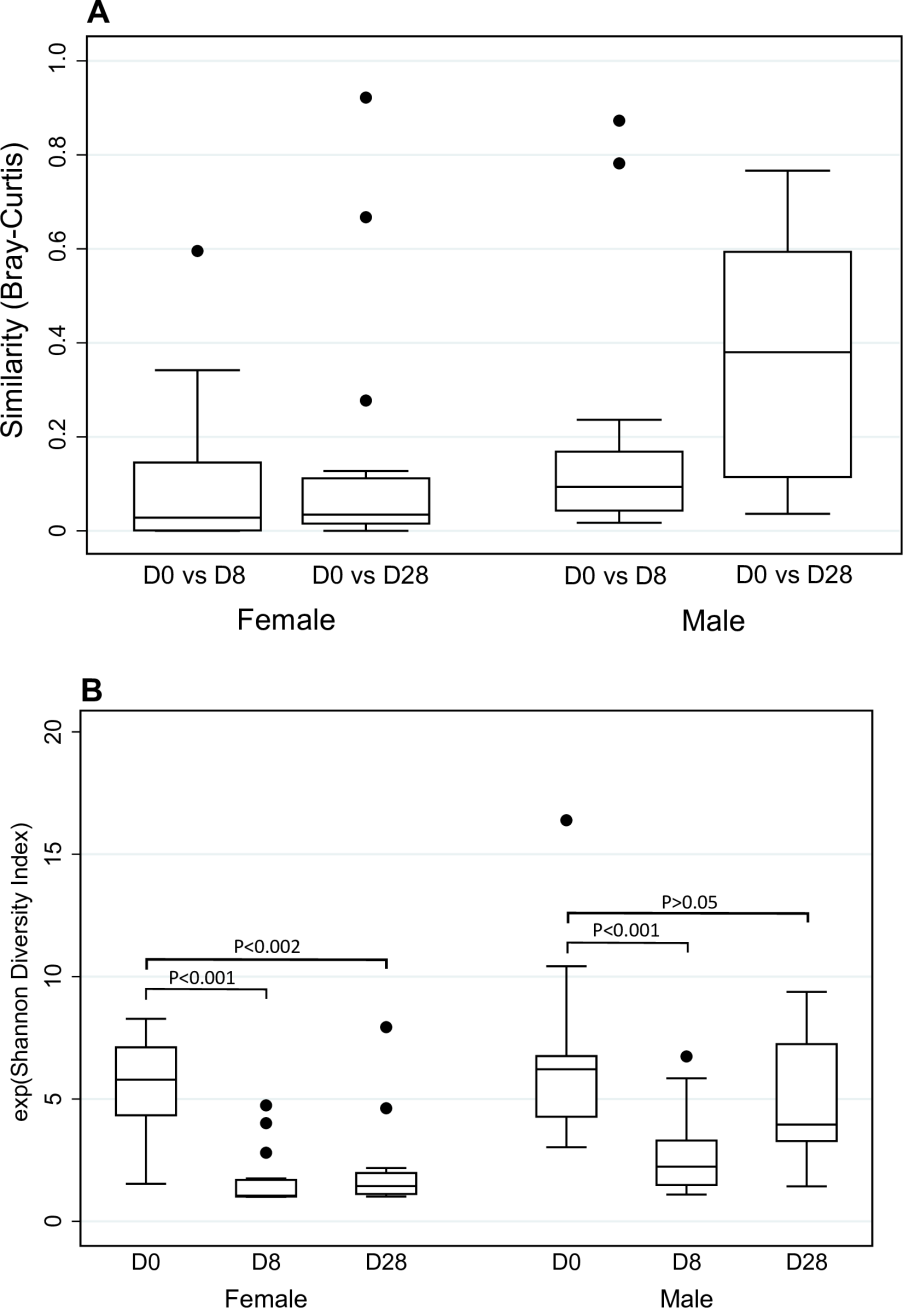
Immediate and sustained effect of dual partner treatment on the composition and diversity of the genital microbiota of females and males. **Panel A.** Effect of treatment on microbiota composition. Bray-Curtis scores were calculated using the between paired specimens from each participant to investigate the change in microbiota composition from baseline to day 8 (D0 vs D8) and baseline to day 28 (D0 vs D28). A lower Bray-Curtis score indicates greater change in microbiota composition. **Panel B.** Effect of treatment on microbiota diversity. Alpha diversity is expressed as effective number of taxa, which is defined as the exponent of the Shannon Diversity Index. Alpha diversity values are presented for specimens collected at baseline (D0), day 8 (D8) and day 28 (D28). Changes in alpha diversity between baseline and day 8 and baseline and day 28 were assessed by the Wilcoxon signed-rank test. Box and whisker plots show median, Interquartile range (IQR), and the most extreme values within 1.5 IQR of the nearest quartile (dots indicate outliers).

The composition of the vaginal microbiota was highly dissimilar immediately post treatment (i.e. day 8) compared to baseline (median Bray-Curtis score of 0.03 [IQR, 0–0.15]), and remained highly dissimilar to baseline at day 28 (0.03 [0.02–0.11]) (Fig 5A). The effective number of taxa (e^H^) in the vaginal microbiota was significantly lower immediately post treatment (median e^H^ = 5.8 [IQR, 4.3–7.1] at day 0 vs e^H^ = 1.0[1.0–1.7] at day 8; p = 0.0005), and remained significantly lower from baseline at day 28 (e^H^ = 1.4 [1.1–2.0]; p = 0.0016) (Fig 5B).

#### Immediate and sustained effect of treatment on the cutaneous penile microbiota

The composition of the cutaneous penile microbiota of individuals was dissimilar immediately post treatment (i.e. day 8) compared to baseline (median Bray-Curtis score of 0.09 IQR, [0.04–0.17]), but became more similar to baseline by day 28 (0.38 [IQR, 0.11–0.59]) (Fig 5A). The effective number of taxa in the cutaneous penile microbiota was significantly lower immediately post treatment (median e^H^ = 6.2 [IQR, 4.3–6.8] at day 0 vs e^H^ = 2.2 [1.5–3.3] at day 8; p = 0.0008), but was only marginally lower in specimens collected at day 28 (e^H^ = 4.0 [3.3–7.2]; p = 0.3) (Fig 5B).

#### Effect of treatment on the prevalence and abundance of key BV-associated bacteria in the vaginal microbiota

Day 0 –day 8: The prevalence of twelve vaginal genera significantly decreased immediately post treatment (q<0.05) (Table 5). The largest decreases were observed for known BV-associated bacteria including *Atopobium*, *Enterohabdus*, *Prevotella*, *Sneathia*, *Anaerococcus* (decrease in prevalence of between 71–82% from baseline). The most prevalent taxon in the vagina immediately post-treatment was *L*. *iners* (detected in 13 of 17 women [76%]).

**Table 5:** Prevalence and changes in prevalence of the 30 most abundant taxa in vaginal specimens over the study period.

	Prevalence in group (n[%])			ΔPrevalence (%)			
Bacterial taxa	Baseline (Day 0, N = 17)^a^	Post antibiotic (Day 8, N = 17)^a^	Study endpoint (Day 28, N = 16)^a^	Day 0–8 ^b^	q value ^c^	Day 0–28^b^	q value ^c^
*Gardnerella*	16 (94)	5 (29)	12 (75)	-65	**0.023**	-19	0.75
*Atopobium*	15 (88)	1 (6)	1 (6)	-82	**0.006**	-81	**0.007**
*Prevotella*	15 (88)	3 (18)	5 (31)	-71	**0.007**	-56	**0.023**
*Dialister*	15 (88)	5 (29)	4 (25)	-59	**0.036**	-63	**0.016**
*Enterorhabdus*	14 (82)	0 (0)	1 (6)	-82	**0.006**	-75	**0.007**
*Sneathia*	13 (76)	1 (6)	0 (0)	-71	**0.007**	-75	**0.007**
*Lactobacillus iners*	13 (76)	13 (76)	12 (75)	0	1	0	1
*Anaerococcus*	12 (71)	0 (0)	7 (44)	-71	**0.007**	-31	0.197
*Parvimonas*	11 (65)	0 (0)	0 (0)	-65	**0.012**	-63	**0.016**
*Megasphaera*	11 (65)	1 (6)	0 (0)	-59	**0.016**	-63	**0.016**
*Fastidiosipila*	10 (59)	1 (6)	0 (0)	-53	**0.023**	-56	**0.023**
*Aerococcus*	10 (59)	5 (29)	4 (25)	-29	0.459	-38	0.333
*Finegoldia*	9 (53)	1 (6)	7 (44)	-47	**0.037**	-13	1
*Gemella*	8 (47)	0 (0)	1 (6)	-47	**0.037**	-44	0.151
*Peptostreptococcus*	7 (41)	0 (0)	1 (6)	-41	0.069	-38	0.13
*Porphyromonas*	6 (35)	0 (0)	1 (6)	-35	0.13	-31	0.197
*Veillonella*	5 (29)	0 (0)	2 (13)	-29	0.197	-19	0.877
*Fusobacterium*	4 (24)	0 (0)	0 (0)	-24	0.333	-25	0.333
*Lactobacillus crispatus*	3 (18)	2 (12)	3 (19)	-6	1	0	1
*Lactobacillus fornicalis*	3 (18)	3 (18)	3 (19)	0	1	0	1
*Corynebacterium*	3 (18)	4 (24)	8 (50)	6	1	31	0.459
*Streptococcus*	2 (12)	3 (18)	5 (31)	6	1	19	0.75
*Shuttleworthia*	1 (6)	0 (0)	0 (0)	-6	1	0	1
*Candidate division TM7*	1 (6)	1 (6)	0 (0)	0	1	-6	1
*Staphylococcus*	0 (0)	3 (18)	4 (25)	18	0.545	25	0.333
*Kluyvera*	0 (0)	1 (6)	0 (0)	6	1	0	1
*Bacillales other*	0 (0)	2 (12)	3 (19)	12	0.882	19	0.545
*Lactobacillus gasseri*	0 (0)	2 (12)	3 (19)	12	0.882	19	0.545
*Enterobacter*	0 (0)	3 (18)	2 (13)	18	0.545	13	0.882
*Escherichia/Shigella*^d^	0 (0)	4 (24)	3 (19)	24	0.333	19	0.545

^a^ Seventeen women provided vaginal specimens for day 0 and 8 paired comparisons, and 16 provided vaginal specimens for day 0 and 28 paired comparisons.

^b^ Change in prevalence (i.e. ΔPrevalence) was calculated using presence absence data for paired specimens i.e. 17 women were analysed for ΔPrevalence day 0–8 and 16 women were analysed for ΔPrevalence day 0–28. ΔPrevalence is expressed as a percentage; a negative ΔPrevalence indicates that the prevalence decreased between visits, while a positive ΔPrevalence indicates that the prevalence increased between visits.

^c^ False discovery rate corrected p-value for change in prevalence as assessed by McNemar’s chi-squared test. Q-value <0.05 are bolded.

^d^ *Escherichia* and *Shigella* cannot be reliably distinguished by their 16S rRNA gene. As such, they are combined here as one taxon *Escherichia/Shigella*.

The abundance of thirteen genera significantly decreased immediately post treatment (S3 Table). Decreases in abundance were observed for *Atopobium*, *Prevotella*, *Enterohabdus*, *Dialister*, *Sneathia*, *Megasphaera*, *Anaerococcus* and *Parvimonas* (q = 0.01). *L*. *iners* was the most abundant bacteria at day 8 and was the only taxon to significantly increase in abundance immediately post treatment (median baseline abundance of 1.9% [IQR, 0.1–7.4%] vs 97.5% [7.9–99.8%] at day 8, q = 0.03).

Day 0 –day 28: The most prevalent taxa in the vagina at day 28 were *L*. *iners* and *Gardnerella* (detected in 12 of 16 women [75%]) (Table 5). Eight of the twelve genera that decreased in prevalence immediately post-treatment in vaginal specimens remained significantly decreased from baseline at day 28, including *Atopobium*, *Enterohabdus* and *Sneathia*.

*L*. *iners* remained the most abundant taxon at day 28, significantly higher than baseline (median day 28 abundance of 91.6% [IQR, 2.6–97.9%], q = 0.02) (S3 Table). The abundance of nine genera remained significantly decreased in vaginal specimens at day 28 compared to baseline. While the prevalence of *Gardnerella* at day 28 was not significantly different from baseline, the median abundance had decreased from 31.7% [IQR, 25.7–40.6%] to 0.1% [0.1–1.9%] (q = 0.012). Interestingly, one woman who had a high abundance of *L*. *iners* at baseline developed an *L*. *crispatus* dominated microbiota at day 8 that was sustained at day 28.

#### Effect of treatment on the prevalence and abundance of key BV-associated bacteria in the cutaneous penile microbiota

Day 0 –day 8: The prevalence of five genera significantly decreased in cutaneous penile specimens immediately post treatment (q<0.05) (Table 6). The most significant reductions were observed for *Finegoldia*, *Peptoniphilus* and *Anaerococcus*, which experienced decreases in prevalence between 68 and 75%. *Corynebacterium* was detected in all males and was the most prevalent taxon in the cutaneous penile microbiota immediately post treatment. *Staphylococcus* was also highly prevalent at day 8 (detected in 14 of 16 males).

**Table 6:** Prevalence and changes in prevalence of the 30 most abundant taxa in penile skin specimens over the study period.

	Prevalence in group (n[%])			ΔPrevalence (%)			
Bacterial taxa	Baseline (Day 0, N = 16)^a^	Post antibiotic (Day 8, N = 16)^a^^,^^b^	Study endpoint (Day 28, N = 15)^a^	Day 0–8 ^c^	q value ^d^	Day 0–28^c^	q value ^d^
*Corynebacterium*	16 (100)	16 (100)	15 (100)	0	1	0	1
*Peptoniphilus*	14 (88)	2 (13)	11 (73)	-75	**0.016**	-14	1
*Finegoldia*	13 (81)	1 (6)	12 (80)	-75	**0.007**	0	1
*Anaerococcus*	13 (81)	2 (13)	13 (87)	-68	**0.023**	7	1
*Staphylococcus*	13 (81)	14 (88)	13 (87)	7	1	6	1
*Prevotella*	12 (75)	5 (31)	10 (67)	-44	0.069	-13	1
*Actinomyces*	11 (69)	4 (25)	11 (73)	-44	0.151	0	1
*Streptococcus*	10 (63)	5 (31)	9 (60)	-32	0.333	0	1
*Dialister*	9 (56)	1 (6)	10 (67)	-50	**0.037**	7	1
*Porphyromonas*	9 (56)	1 (6)	4 (27)	-50	**0.037**	-33	0.197
*Micrococcus*	7 (44)	3 (19)	2 (13)	-25	0.333	-34	0.197
*Gardnerella*	6 (38)	4 (25)	4 (27)	-13	1	-6	1
*Peptostreptococcus*	5 (31)	0 (0)	3 (20)	-31	0.197	-13	1
*Campylobacter*	5 (31)	0 (0)	2 (13)	-31	0.197	-20	0.75
*Veillonella*	5 (31)	3 (19)	4 (27)	-12	1	-6	1
*Corynebacteriaceae_other*	5 (31)	3 (19)	5 (33)	-12	1	0	1
*Lactobacillus_iners*	5 (31)	3 (19)	4 (27)	-12	1	-6	1
*Bacillales_other*	5 (31)	5 (31)	7 (47)	0	1	20	0.877
*Gemella*	4 (25)	1 (6)	2 (13)	-19	0.545	-14	1
*Enhydrobacter*	4 (25)	2 (13)	2 (13)	-12	1	-7	1
*Brevundimonas*	4 (25)	3 (19)	2 (13)	-6	1	-7	1
*Neisseria*	3 (19)	0 (0)	2 (13)	-19	0.545	-7	1
*Rothia*	3 (19)	1 (6)	2 (13)	-13	1	-6	1
*Haemophilus*	3 (19)	2 (13)	2 (13)	-6	1	-7	1
*Enterobacteriaceae_otu1*	3 (19)	3 (19)	0 (0)	0	1	-20	0.545
*Zimmermannella*	2 (13)	1 (6)	1 (7)	-7	1	-6	1
*Escherichia/Shigella*^e^	2 (13)	4 (25)	1 (7)	12	0.882	-7	1
*Pseudomonas*	2 (13)	5 (31)	0 (0)	18	0.75	-13	0.882
*Enterobacter*	1 (6)	3 (19)	0 (0)	13	0.882	-7	1
*Stenotrophomonas*	1 (6)	4 (25)	3 (20)	19	0.75	13	1

Abbreviations: otu, operational taxonomic unit.

^a^ Sixteen males provided penile specimens for day 0 and 8 paired comparisons, and 15 provided penile specimens for day 0 and 28 paired comparisons.

^b^ Two day 8 penile specimens failed to meet the sequence depth threshold and were substituted with day 14 specimens

^c^ Change in prevalence (i.e. ΔPrevalence) was calculated using presence absence data for paired specimens i.e. 16 males were analysed for ΔPrevalence day 0–8 and 15 males were analysed for ΔPrevalence day 0–28. ΔPrevalence is expressed as a percentage; a negative ΔPrevalence indicates that the prevalence decreased between visits, while a positive ΔPrevalence indicates that the prevalence increased between visits.

^d^ False discovery rate corrected p-value for change in prevalence as assessed by McNemar’s chi-squared test. Q-value <0.05 are bolded.

^e^ *Escherichia* and *Shigella* cannot be reliably distinguished by their 16S rRNA gene. As such, they are combined here as one taxon *Escherichia/Shigella*.

The abundance of *Prevotella*, *Peptoniphilus*, *Finegoldia*, *Dialister* and *Anaerococcus* was significantly reduced in cutaneous penile specimens collected immediately post treatment (q<0.05) (S3 Table). *Staphylococcus* and *Corynebacterium* were the most abundant genera in males immediately post-treatment (median abundance of 14.8% [IQR, 0.4–72.5%] and 13.9% [1.9–31.5%], respectively).

Day 0 –day 28: *Corynebacterium* was the most prevalent and most abundant genus in cutaneous penile specimens at day 28 (detected in all specimens at a median abundance of 22.6% [IQR, 13.3–41.4%]) (Tables 6 and S3). No significant changes in the abundance or prevalence of specific penile skin bacteria were observed between baseline and day 28, suggesting that the BV-associated bacteria which had decreased immediately post-treatment had returned to pre-treatment levels by day 28.

Given the small number of circumcised males (n = 4), there were insufficient specimens to examine the impact of circumcision on the cutaneous penile microbiota pre- and post-treatment.

#### Effect of treatment on the total bacterial load of the vaginal and penile microbiota

Despite the observed reduction in effective number of species immediately post treatment in men and women there were no observed trends in bacterial load following treatment (S1 File), indicating overall load of bacteria was maintained despite the decreased diversity. As expected, the total bacterial load was lower in urine specimens compared to vaginal and cutaneous penile specimens (S1 File).

#### Impact of sexual partnerships on the similarity of vaginal and cutaneous penile microbiota

The impact of sexual partnership on the genital microbiota of couples was assessed for 20 couples at baseline, 16 couples at day 8 and 15 couples at day 28 (Fig 2).

Unexpectedly, the vaginal microbiota of a female was not more similar to the cutaneous penile microbiota of their sexual partner, when compared to non-partner males, either at baseline or longitudinally (S2 Fig); no comparison with the urethral microbiota could be made for reasons previously outlined. Prevalent taxa in the vaginal and cutaneous penile microbiota of partners showed weak to moderate correlations at baseline and day 8 (S4 Table). At day 28, *Dialister* and *Prevotella* were strongly positively correlated between the vaginal and cutaneous penile microbiota of sexual partners (*ρ* = 0.72 and *ρ* = 0.71, respectively) with borderline significance (q = 0.05; S4 Table). No other correlations were significant.

Following treatment, all couples resumed unprotected sexual intercourse and all except one couple had resumed oral sex; two couples had anal sex following treatment. We were therefore unable to investigate the effect of specific sexual practices on the genital microbiota of couples.

### BV recurrence

At day 8, no female had BV recurrence by Nugent score. There were two cases of recurrence recorded. One couple had frequent unprotected sex throughout the treatment period and BV recurred at day 14 (NS = 8). Another female experienced recurrence at day 21 (NS = 8) following incomplete adherence to treatment and the resumption of unprotected sex at day 14. Women who did not recur during the study had a predominance of *L*. *iners* following treatment. In contrast, *L*. *iners* was not detected in the vaginal microbiota of the two women who recurred at any time post treatment.

## Discussion

This pilot study aimed to determine whether combined oral and topical antimicrobial treatment of male partners of women with BV was acceptable and well tolerated, and to examine the effect of dual-partner therapy on the vaginal and penile microbiota. Treatment of males was well tolerated and adherence was high, which indicates high acceptability of the therapy to males. In addition, treatment resulted in changes to both vaginal and cutaneous penile microbiota. Immediately post completion of treatment, there was a significant reduction in the prevalence and abundance of BV-associated bacteria in the vaginal microbiota and a shift to a low diversity and often *L*. *iners* dominated environment. Importantly, the suppression of BV-associated bacteria was sustained at three weeks post treatment in the majority of women. BV-associated bacteria were abundant in the penile skin of male partners of women with BV and interestingly, there was a significant reduction in bacterial diversity and a depletion of BV-associated bacteria in the cutaneous penile microbiota immediately post treatment, similar to what has been observed in trials of male circumcision[32, 56]. However, re-emergence of BV-associated bacteria in the cutaneous penile microbiota three weeks post treatment was common. This pilot study was not powered to look at the impact of dual-partner treatment on recurrence, however BV recurrence was uncommon. Our data provide an evidence base for the development of larger trials that have extended follow-up, sample the urethral microbiota, and a randomly allocated placebo or non-treatment comparator.

This study evaluated male treatment with a combination of oral metronidazole and topical penile application of clindamycin. There was a strong willingness of males to receive treatment, with over 90% of approached males agreeing to participate in this study. This is higher than the 70% participation rate reported by Mengel et al[26], but is consistent with an RCT of a topical microbicide for male-partners of women with BV[57]. Women were asked at screening if they thought it likely that their male partner would agree to be involved which is likely to have biased the study towards a higher participation rate. Retention rates were reasonable with only five couples providing incomplete sets of data (four LTFU and one withdrew due to break up). Self-reported adherence to both study treatments was high, with the majority of participants taking all doses of medication. Male adherence to metronidazole was higher than adherence to clindamycin, which could suggest a preference for oral treatment over topical treatment.

We chose combination antimicrobial therapy for males given recent literature on the composition of the microbiota of the male genital tract in male partners of women with BV[22, 23, 32], and we hypothesised that the combined spectrum of activity of the two antibiotics would achieve broad activity against the range of BV-associated bacteria in urethral and cutaneous sites. In addition, oral metronidazole and vaginal clindamycin have equivalent four week cure rates for BV[58]. Both treatments were well tolerated by males, with minimal side effects reported. This is particularly important for topical clindamycin which is not licensed for use in males. Metronidazole is widely used in females and males and the male who experienced a mild truncal rash may have had an allergy to metronidazole, which is known to be a rare side effect occurring in < 0.1% the population[59].

In addition to assessment of acceptability and tolerability, we assessed the vaginal and penile microbiota of couples at baseline, post-treatment (day 8) and at one month (day 28). The immediate and marked response of the vaginal microbiota to antibiotic treatment observed in this study is consistent with research by other groups[60–62]. The observed sustained suppression of BV-associated bacteria and low abundance of *Gardnerella* in women three weeks post treatment is encouraging, but without a control group of untreated males we are unable to determine if this is attributable to the additional benefit from concurrent male partner treatment. Previous investigations of the vaginal microbiota have provided varying reports regarding the re-emergence of BV-associated bacteria following treatment of women only. Mayer et al [60] and Ravel et al [61] reported that return to a pre-treatment microbiota and re-emergence of BV-associated bacteria was common occurring within three to four weeks of treatment. Conversely, other authors have shown sustained reduction in bacterial diversity and/or BV-associated bacteria following treatment with metronidazole [62–64]. For example, Gottschick et al [64] reported a low diversity, lactobacilli dominated vaginal microbiota at 7 to 28 days post treatment with oral metronidazole that was sustained in a majority of women at up to 14 weeks post treatment. Similarly, Xiao et al [62] followed 65 women after treatment with intravaginal metronidazole gel and reported a reduction in bacterial diversity that was sustained at 30 days post treatment. Both Gottschick et al and Xiao et al reported a similar percent of women cured, 72 and 74% respectively[62, 64]. Women who did not recur during our study had a predominance of *L*. *iners* following treatment, whereas the two women who recurred did not. These data suggest that a rapid increase in lactobacilli post treatment may be necessary for sustained effectiveness of treatment and prevention against reinfection. However, a protective role of *L*. *iners* against BV has not been established and *L*. *iners* is frequently detected in women with BV, without BV and in the vagina following antimicrobial therapy for BV[7, 8, 60, 61, 64–66]. This has led some groups to suggest that both pathogenic and beneficial strains of *L*. *iners* may exist, while others suggest that *L*. *iners* acts as a transitional species, bridging the gap between a diseased state and a healthy state [8, 60, 67–69].

The immediate effect of antibiotic treatment on the cutaneous penile microbiota we observed can be likened to the previously described effect of circumcision [32, 56]. There was a dramatic change, specifically a reduction in bacterial diversity and a depletion of anaerobic BV-associated bacteria including *Peptoniphilus*, *Finegoldia* and *Dialister*. Re-emergence of anaerobic BV-associated bacteria occurred in the cutaneous penile microbiota of most males within three weeks of ceasing antibiotics. Interestingly, this didn’t equate temporally to a re-emergence of BV-associated bacteria in the vaginal microbiota of their sexual partner in all participants. This raises the question: where are these organisms coming from, if not from the female partner? It is possible that BV-associated bacteria remain below the level of detection on the penile skin, reside in the prostate [70, 71], or persist in the urethra (which could not be measured in this study), following treatment and then proliferate in the absence of ongoing treatment. However, an alternative theory is that BV-associated bacteria are reintroduced to the penile microbiota via the oral cavity during oral sex or via the gastrointestinal tract, which have been considered by various investigators as potential mechanisms for BV acquisition/recurrence [72–74].

The re-emergence of BV-associated bacteria in the cutaneous penile microbiota, but not the vaginal microbiota of sexual partners at day 28 highlights that sexual exchange of genital microbiota is not necessarily easily measured. A key limitation of this dataset was the absence of urethral specimens of sufficient quality to provide data on the abundance and prevalence of specific BV-associated bacteria at the urethral site. Zozaya et al reported that the penile skin and urethral microbiota of male partners of women with BV were more similar to the vaginal microbiota of their sexual partner compared to that of other women with BV, as well as a high level of correlation of specific taxa (primarily BV-associated bacteria) in the genital microbiota of BV-couples[23]. Furthermore, sexual partners have been shown to share the same strains of *G*. *vaginalis* [75]. In our study, we found no difference in the similarity of the vaginal and penile skin microbiota between sexual partners versus non-partners. In addition, while BV-associated bacteria were abundant in males, we found only weak to moderate correlation between the prevalent taxa in the vaginal and cutaneous penile microbiota of partners both pre-treatment and immediately post treatment.

The lack of overall similarity between sexual partners and lack of correlation between specific taxa observed could be explained by the small number of couples enrolled in this pilot study, or due to the study population examined and sampling protocol used. Both the Zozaya[23] and Eren[75] studies utilised clinician collected specimens for genital microbiota characterisation whereas we used self-sampling to optimise participant recruitment and retention; it also allowed for specimen collection at home. Additionally, the majority of women were highly similar at each time point during the study, with a predominance of *Gardnerella* pre-treatment and a predominance of *L*. *iners* post-treatment. These factors, together with the small sample size, may have reduced the discriminatory power of our analysis.

*Gardnerella* was the predominant BV-associated bacteria identified in females at baseline, but was almost absent from cutaneous penile specimens at baseline. It has been hypothesised that colonisation of *G*. *vaginalis* may be limited to the distal urethra in males[76] and Nelson et al previously reported failure to detect *G*. *vaginalis* from coronal sulcus samples of adolescent boys, despite detecting it in 28% of urine samples collected from the same participants[21]. Consistent with this, while we observed a low abundance and prevalence of *Gardnerella* in the cutaneous penile skin of participants throughout the study, our limited urethral data available suggests that *Gardnerella* is both prevalent and abundant in the male urethra prior to treatment. Future studies should utilise collection of high quality urethral specimens to obtain a better understanding of the sharing of *G*. *vaginalis* and genital microbiota between sexual partners. Other organisms may also have a role in BV recurrence. Both *Prevotella* and *Dialister* showed a strong positive correlation between the vaginal and cutaneous penile microbiota of sexual partners at day 28. These organisms have been detected in women with BV [5, 7–9], and the presence of *Prevotella* and *Dialister* in the penile skin (as well as *Mobiluncus* and *Porphyromonas*) has previously been reported as an indicator of female-partner Nugent-BV[22]. Further investigation of the involvement of *Prevotella* and *Dialister* in the development and recurrence of BV is needed, particularly in women who have sex with men.

This pilot study set out to determine acceptability and tolerability of male partner treatment. Although this study was not powered to measure the effect of male partner treatment on BV recurrence, we recorded two cases of BV recurrence (12.5% recurred) in the study population. Overall, this group of women had a substantial risk of BV recurrence as over 80% had a past history of BV, all had a regular sexual partner, and all were having unprotected sex; each of these factors have been associated with an increased risk of BV recurrence in past studies[11, 77].

Current reviews provide support for two key drivers of BV recurrence: persistence of disease and reinfection from a sexual partner ([13, 14, 78]). Presence of a dense biofilm, antibiotic resistant BV-associated bacteria and/or host factors may be particularly important in persistence. In contrast, specific partner factors, such as lack of circumcision, may increase the risk of reinfection. These differing mechanisms may explain why it has been so challenging to improve treatment efficacy and highlight the importance of further studies fully understand the pathogenesis of BV recurrence.

This study has a number of limitations. Approximately one third of the urine swabs collected for urethral microbiota characterisation had low bacterial load and did not meet the sampling depth required for analysis. As detailed above, the limited urethral data creates an incomplete picture of what (and how) bacteria are exchanged through sexual intercourse, and may have particularly affected our ability to compare the genital microbiota of couples and to accurately measure the abundance of specific bacteria in males, namely *Gardnerella*. This is a key limitation given the interest in *G*. *vaginalis* as a key aetiological agent in BV. Future studies should utilise collection of appropriate urethral specimens. Self-collected penile-meatal swabs have been shown to be acceptable for STI testing[79] and would facilitate self-sampling of the urethral microbiota at home.

The primary intention of this pilot study was to assess the acceptability and tolerability of the intervention, for which we found positive outcomes. As this was a pilot, couples were only followed for three weeks post treatment, and due to the small number of participants, we were unable to assess the effect of demographics, adherence or sexual behaviours on genital microbiota composition. Extending follow up for at least another three months may have provided a more complete understanding of the exchange of the genital microbiota between sexual partners, and together with a larger sample size, the true impact of male reinfection on BV recurrence. Two couples were treated for an STI at baseline and seven women reported taking antibiotics in the month prior to enrolment; four of these women reported metronidazole use. In most cases the antibiotic exposure preceded the diagnosis of BV by days to weeks and regarding the two couples treated at baseline, neither azithromycin nor ceftriaxone have strong anaerobic coverage. Importantly, all women had BV at enrolment and although is possible that antibiotic exposure may have impacted on the genital microbiota of these individuals, any ongoing impact on the genital microbiota during the month of follow-up is unlikely. Co-infection of BV with STIs is common and represents a key challenge of recruiting in STI services. We chose not to exclude the participants treated for STIs at baseline given the primary focus of this pilot study was acceptability and tolerability, however larger studies of male partner treatment should impose stricter eligibility criteria. Additionally, seven women reported unprotected vaginal exposure (received oral sex, or had vaginal or anal sex) during the treatment period, despite the instruction of abstention or condom use. Unprotected vaginal exposure during treatment may have impacted the composition of the vaginal and penile microbiota or treatment efficacy, however given the small sample size we were unable to assess this. Finally, this study did not include a placebo or untreated comparator, preventing comparison of dual-partner treatment to treatment of women only.

## Conclusions

We report that male partner treatment for BV is acceptable and treatment was tolerable. Current treatments for BV are sub-optimal and associated with unacceptably high recurrence rates. Despite not being powered to assess the effect of treatment on recurrence, we observed a low incidence of BV recurrence in a group of women with a past history of BV who were having unprotected sex with a regular partner following dual-partner treatment. There is ongoing debate concerning the pathogenesis of BV recurrence but epidemiological and recent microbiological data strongly suggest sexual transmission is integral to both recurrent and incident disease. Our data support the need for high quality studies of male partner treatment with accompanying vaginal, cutaneous penile and urethral microbiota data, an untreated or placebo group and long-term follow-up to define the actual contribution of reinfection and disease persistence to BV recurrence in women. These data support recent reviews that suggest that combination approaches such as dual partner treatment and biofilm-disrupting agents may are likely to be needed to improve treatment outcomes[13, 14].

## Supporting information

S1 File Additional methods and results. (DOCX)

S2 File TREND statement checklist. (DOCX)

S3 File Study protocol. (PDF)

S1 Table Negative controls analysed. (DOCX)

S2 Table Potential contaminants removed from OTU table prior to analysis. (DOCX)

S3 Table Proportional abundances of the 30 most abundant taxa in vaginal specimens and cutaneous penile specimens over the study period. (DOCX)

S4 Table Correlation of specific bacterial taxa between vaginal and cutaneous penile specimens of couples at baseline, day 8 and day 28. (DOCX)

S1 Fig Heatmap of bacterial abundance from urine specimens collected at baseline, day 8 and day 28. Each vertical line represents the bacterial composition of one urine specimen. Only the 30 most abundant taxa found in urine specimens are included in the heatmap. Study day is displayed above the heatmap in red (day 0), blue (day 8) and yellow (day 28).
(PDF)

S2 Fig Comparison of Bray-Curtis Similarity distances between the genital microbiota of sexual partners and non-sexual partners over the study period. Box and whisker plots show median, Interquartile range (IQR), and the most extreme values within 1.5 IQR of the nearest quartile (dots show outliers). A lower Bray-Curtis similarity score indicates the vaginal and penile microbiota of couples is dissimilar. Comparisons are made between specimens from couples collected before treatment (D0), immediately after treatment at day 8 (D8) and three weeks after cessation of treatment at day 28 (D28). Differences in the similarity of bacterial communities of sexual partners compared to non-partners were assessed at each time point using the Wilcoxon signed-rank test.
(PDF)
