# Supplementary material for: Combined oral and topical antimicrobial therapy for male partners of women with bacterial vaginosis: Acceptability, tolerability and impact on the genital microbiota of couples - A pilot study
Source: PLoS One. 2018 Jan 2;13(1):e0190199. doi: 10.1371/journal.pone.0190199 (PMC5749747; doi:10.1371/journal.pone.0190199)
Supplement: S1 File — (DOCX) [file pone.0190199.s001.docx]

**Supplementary Methods**

DNA extraction using phenol/chloroform

Sample (200 µl) was transferred to a sterile 1.5 ml tube, centrifuged (13,000 rcf, 20 min), then supernatant removed. The sample was resuspended in 500 µl of PBS, and SDS and Proteinase K added (final concentrations of 1% w/v and 0.1 mg/mL, respectively). The sample was mixed and incubated at 56°C for 1 hour, then extracted with an equal volume of phenol:chloroform (1:1 mix) by mixing and centrifugation (5 min, 10,000 rcf). The aqueous phase was transferred to a new tube and extracted with an equal volume of chloroform. DNA was precipitated with an equal volume of chilled 100% isopropanol, collected by centrifugation (13,000 xg for 20 minutes), washed with 500 µl 70% ethanol, and air dried. DNA was resuspended in 100 µl of TE buffer.

**Supplementary Results**

Total bacterial load differed between specimen types, but treatment does not affect the total bacterial load of the vaginal or penile microbiota

Bacterial loads were assessed to determine the effect of antibiotic treatment on total bacterial content. As expected, the total bacterial load was lower in urine specimens (mean 7.1 [standard deviation{SD}=4.7] log_10_ copies/5 µL) compared to both penile (9.7 [SD=3.2] log_10_ copies/5 µL, p=0.01[95%CI:-4.6, -2.7]) and vaginal specimens (15.4 [SD=1.3] log_10_ copies/5 µL, p<0.001[95%CI:-10.2,-6.5]).

Furthermore, there were no observed trends in bacterial load following treatment in either vaginal or penile specimens. Mean total bacterial load at baseline in vaginal specimens was 15.5(SD=1.1) log10 copies/5 µL at baseline, compared to 15.3 (SD=1.5) log_10_copies/5 µL at day 8, and 15.4 (SD=1.4) log_10_copies/5 µL at day 28 (p=0.6[95%CI:-0.7, 1.1] and p=0.9[95%CI:-0.9, 1.0], respectively). Mean total bacterial load at baseline in penile specimens was 9.7(SD=2.8) log10 copies/5 µL at baseline, compared to 9.5 (SD=3.4) log_10_copies/5 µL at day 8, and 10.4 (SD=2.8) log_10_copies/5 µL at day 28 (p=0.9[95%CI:-2.4, 2.7] and p=0.3[95%CI:-2.7, 1.0], respectively).
