# Supplementary material for: Combined oral and topical antimicrobial therapy for male partners of women with bacterial vaginosis: Acceptability, tolerability and impact on the genital microbiota of couples - A pilot study
Source: PLoS One. 2018 Jan 2;13(1):e0190199. doi: 10.1371/journal.pone.0190199 (PMC5749747; doi:10.1371/journal.pone.0190199)
Supplement: S3 File — (PDF) [file pone.0190199.s003.pdf]

StepUp - Pilot study to determine the feasibility and acceptability of male partner treatment in women with bacterial vagnosis (BV).

Background

Bacterial vaginosis (BV) is the most common vaginal infection in reproductive age women and is associated with serious and costly sequelae. Prevalence estimates of 29% in North-American women[1], 12% in non-Indigenous and 30% in Indigenous Australian women[2], and >50% in women in East/Southern Africa[3] have been reported. BV increases women's risk of acquiring and transmitting HIV[4,5] and is associated with an increase in pre-term delivery, low birth weight, miscarriage and PID[6]. The population attributable risk of BV for pre-term delivery in 2002 in the US was estimated to be 30% at a cost of USD 1 billion per annum[6]. Over 50% of women with BV experience symptoms, with qualitative studies by our group showing BV is associated with moderate-severe impact on self-esteem, sexual relationships and quality of life[7].

The aetiology and pathogenesis of BV is unclear. No single species is universally present in BV and absent in the "healthy" vaginal microbiota. Instead BV is characterised by depletion of key *Lactobacillus* spp., primarily *L.crispatus,* and increased loads of commensal anaerobes including *G.vaginalis*, *A.vaginae* and other non-cultivatable BVAB such as *Megasphaera*, *Prevotella spp.*, *Sneathia* and *Clostridiales* spp.[8]. Many BVAB have evolved to exist in humans only, so a reliable BV animal model does not exist, hindering efforts to investigate its pathogenesis and aetiology. Next-generation sequencing has advanced our understanding of this complex polymicrobial condition with high diversity and loads of bacterial species reported in women with BV compared to healthy controls[8]. However, it is not known if BV results from transmission of a polymicrobial consortium or acquisition of a single founder organism. Two of the commonest BVAB, *G.vaginalis* and *A.vaginae*, appear to dominate a vaginal wall biofilm that has been associated with BV[9].

Current recommended BV treatment is associated with unacceptably high recurrence rates Recommended BV treatment involves oral or vaginal metronidazole (MTZ) or clindamycin. These agents have broad anaerobic cover and equivalent one month cure rates of 70-80%[10]. Importantly, they do not achieve sustained high levels of cure, with recurrence rates of 52% within 6 months reported by our group[11]. High sustained cure rates have not been achieved with any published treatment approaches to date, including longer course suppressive antibiotic regimens, combination antibiotic and antibiotic-probiotic regimens[12]. Given the high global burden and significant morbidity associated with BV, there is a pressing need to identify new approaches to improve cure rates and reduce sequelae.

We have shown that BV is highly likely to be sexually transmitted and that reinfection from untreated sexual partners may be responsible for the high rates of BV recurrence. The concept that BV may be sexually transmitted has come in and out of favour over the last 50 years and remains controversial. Difficulties encountered in determining its aetiology, absence of a clear disease counterpart in males, and failure of 5 of 6 male partner treatment trials to impact on recurrence[13,14], have all been significant impediments in determining whether BV is sexually transmitted. A recent systematic review of these male partner treatment trials concluded that a number of factors including insufficient power, use of non-standard BV treatment regimens including single dose regimens, no measures of adherence, and poor retention rendered the findings inconclusive by current standards and recommended larger trials

using recommended first line therapies be conducted.[14]. In support of sexual transmission of BV, there are published data to indicate that the long-term poor performance of recommended therapies may at least partly be attributed to post-treatment sexual behaviours. Women exposed to an ongoing male partner following BV treatment have a two fold increased risk of recurrence after adjusting for sexual frequency, condom use and hormonal contraception in two studies[11,15]. Meta-analysis has also shown that consistent condom use is associated with reduced odds for BV (0.8; 95% CI 0.8-0.9)[16], and several but not all studies have found inconsistent condom use for penile-vaginal sex is associated with BV-recurrence following treatment[15,17,18]. Male carriage of *G.vaginalis*, an organism considered by some investigators to be integral to the development of BV[19], is commonly reported[20-22], and pyrosequencing of the microbiota of the coronal sulcus and distal-urethra in male adolescents[23], showed these sites to be colonised by BVAB, with the composition of the sulcus microbiota significantly influenced by circumcision and sexual activity[23]. In one prospective study male circumcision was associated with a significant reduction in penile anaerobic microbial flora, including BV-associated genera Clostridiales and Prevotellaceae[24]. Furthermore a secondary analysis within a Ugandan circumcision trial showed wives of circumcised males had a significantly reduced risk of BV (adjPRR=0.60; 95%CI=0.38-0.94) compared to wives of uncircumcised males[25]. While there are fewer studies examining BV-associated biofilm in men, biofilm has been detected in male urine and semen and more commonly found in the male partners of females with BV than healthy controls [26]. Collectively, published epidemiological data provides broad support for sexual transmission of BV between men and women, and highlights the need to conduct sufficiently powered male partner treatment trials to determine if this strategy reduces BV recurrence and associated sequelae.

Research Plan

Overall Objective To determine the feasibility and acceptability of male partner treatment in women with BV, and to examine the impact of dual partner treatment on BV-associated bacteria in the male and female genitalia following treatment.

Rationale BV is common, associated with serious sequelae and current recommended antibiotic therapy is associated with unacceptably high recurrence. Meta-analysis of observational data provides strong evidence for sexual transmission of BV, and studies of recurrent BV indicate that reinfection from sexual partners is contributing to the high rates of recurrence but this requires evaluation by RCT. A number of investigators have shown that BV-associated bacteria (BVAB) are present in male partners of women with BV on the penile skin and also distal urethra indicating that both oral and topical therapy may be required to achieve clearance of BVAB. Both oral metronidazole and topical clindamycin are used as recommended first line agents in women with BV for 7 days and have equivalent cure rates, no male treatment trials have evaluated topical and oral combination therapy in males. Prior male partner treatment trials have relied on oral antibiotics alone, and several only evaluated single dose therapies, which may have contributed to the failure of a number of these trials. Pilot data is required to obtain evidence for the feasibility and acceptability of both oral and topical antibiotic therapy in male partners of women with BV and evidence that antibiotic therapy use in male partners is associated with a reduction in BV-associated bacteria in males using qPCR. Oral metronidazole is a commonly used antibiotic in men and women being prescribed for amoebiasis, trichomoniasis and anaerobic bacterial infections, including bacterial vaginosis in women. Its safety

profile is well described and there is a long history of use in both genders. Topical clindamycin cream is only indicated for treatment of bacterial vaginosis in women and is known to weaken condoms, however sexual activity during its use in women is not contraindicated and men therefore get exposed to this agent during sexual intercourse. Both agents will be prescribed in men outside the licensed indications. It is not anticipated that men will experience significant side effects from either agent however data on cutaneous reactions such as irritation, redness and itch and general tolerability and acceptability data will be collected. These acceptability, feasibility and microbiological data are required to inform the design of a multi-centre double-blinded placebo-controlled male partner treatment trial which would form the basis of a category 1 grant ie. NHMRC.

Approach

Study design: All female participants will receive oral metronidazole (MTZ) 400 mg twice daily for 7 days for treatment of BV, in keeping with recommended clinical practice. If MTZ is contraindicated, a 7-day regimen of topical vaginal 2% clindamycin will be prescribed, in accordance with international treatment guidelines.

Male partners of female participants will receive oral metronidazole 400mg twice daily and topical 2% clindamycin cream to be applied to the head of the penis and upper shaft (under the foreskin if uncircumcised) twice daily for 7 days.

Sampling frame: Women will be recruited from Melbourne Sexual Health Centre (MSHC) MSHC is the largest sexual health service in Victoria and diagnoses over 550 cases of BV annually. Men will be recruited through their female partners who have been diagnosed with BV at MSHC.

Inclusion criteria:

Women will be eligible if they*:*

1. are 18-55 years of age
2. have symptomatic BV, microbiologically defined as a Nugent score of 4-10 and 3-4 Amsel criteria*,
3. are willing and able to comply with protocol requirements
4. have a regular male partner who is willing to be enrolled in the trial.

*The Amsel method is an established BV diagnostic method where BV is diagnosed if ≥3 of 4 Amsel criteria are present: i) vaginal pH>4.5, ii) characteristic vaginal discharge, iii) positive amine test and iv) Clue cells on microscopy[27]. The Nugent method, also an established diagnostic method, is commonly used in clinical trials and scores vaginal bacterial morphotypes on Gram stain[28]. A Nugent score (NS) of 0-3 represents a "healthy" *Lactobacillus* spp. dominant state, 4-6 an intermediate/ transitional state, and 7-10 is diagnostic of BV, with abundant anaerobes and scarce/absent lactobacilli. A combination of 3-4 Amsel criteria and NS=4-10 is commonly used in clinical trials to diagnose symptomatic BV.

Women will be ineligible if they:

1. are HIV positive
2. are pregnant or breast feeding
3. are diagnosed with current PID
4. have an allergy to both first line antibiotics for BV, MTZ and clindamycin
5. have other current sexual partners

Men will be eligible if

1. their regular female partner meets the eligibility criteria

2. they are willing and able to comply with protocol requirements

Men will be ineligible if they:

1. report a drug allergy to MTZ or clindamycin,
2. are HIV positive
3. have other current sexual partners

Primary aim: To determine the acceptability and feasibility of male partner treatment in women with BV

Secondary aims:
To determine if dual partner treatment for BV is associated with reduction in load of key BV-associated bacteria (BVAB) in the male and female genital tract over one month following treatment.

Study Outcome: Acceptability and feasibility of male partner treatment

Recruitment and Study-related Procedures
Women attending MSHC with vaginal symptoms routinely undergo a clinical history and vaginal examination, pH estimation (Spezialindikator strips pH 2-9, Merck & Co, USA), collection of genital specimens for microscopy, *C.trachomatis* PCR, *N.gonorrhoeae* and *T.vaginalis* culture, and assessment for BV by Nugent and Amsel methods. BV is routinely treated with oral metronidazole 400mg twice daily for 7 days (current first line treatment), or if not previously tolerated or contraindicated topical vaginal clindamycin cream daily for 7 days. The treating clinician and pharmacist instruct participants regarding antibiotic use and potential side effects. Clinicians at MSHC, who are specialist sexual health physicians, are highly experienced in recruiting for BV trials having recruited 2400 women into clinical studies of BV. A study alert activates when a diagnosis of BV is entered into the patient computerised record to remind clinicians to discuss the study and call the research nurse.

Enrolment visit (Day (D) 0): Women diagnosed with BV and who report a current regular male partner and are interested in participating will be referred to the research nurse who will explain the study and assess eligibility. Women who believe it is likely that their male partner will agree to be involved, will be recruited and informed consent obtained. Female participants will be asked to complete a questionnaire on sexual and contraceptive behaviours, BV symptomatology and past episodes, and demographic information, and to self-collect two high-vaginal swabs for investigation of vaginal bacterial species by molecular methods.

Women who enrol will be asked if it is possible to contact their partner at the time of recruitment. There will be three pathways for male recruitment:

1. *In clinic (principle method of recruitment)*: Male partners will be spoken to over the phone by the research nurse while their female partner is in the clinic and if he agrees an appointment will be made with the nurse within 7 days. The study procedures will be explained, written informed consent and a penile skin swab and urine sample will be obtained and a questionnaire completed. At this visit the male will be provided with the antibiotics and home test packs. Men will be asked not to have unprotected sex with their female partner until both of them have completed the one week course of antibiotics. Men will be asked to collect a penile skin and urine sample at day 8, day 14, day 21 and

day 28 and to complete a brief questionnaire on each occasion. Samples and questionnaires will be returned by post to investigators.

2. *Phone consult*: If the male partner is unable to attend an appointment within 7 days but is willing to make a time for a formal phone consultation a time will be made for a phone consultation with the study clinician and nurse in which the study procedures will be explained in detail. Phone consultations have been used for other approved STI testing and treatment programmes at MSHC (ie Testme). A clinical consultation will be created in the MSHC electronic record. The purpose of the study and all study procedures will be clearly explained, current and relevant past medical issues and presence of drug allergies and adverse drug reactions will be identified and documented. If happy to proceed male participants will be posted a study pack containing a patient information and consent form, questionnaire, instructions for self-collection of penile skin and urine samples, and study medication. Completed and signed consent forms, questionnaires and samples will be returned to investigators by post in a reply paid envelope. Men will be asked not to have unprotected sex with their female partner until both of them have completed the one week course of antibiotics.

3. *Unable to be contacted on the day*: Female participants who believe their partner is highly likely to agree to participate will be sent home with a study information pack for their male partner that provides information about BV, about what is involved if they participate in the study, a participant information and consent form, self-sampling instructions, genital swab and urine swab home test packs and a reply paid envelope. Women will be asked to discuss participation in the study with their partner and for him to call the study team on the free number provided, if he is willing to participate. A research team member will explain the study in detail over the phone to the male partner and men will be given the option of attending for an appointment or to have a phone consultation as outlined above. Study related medications will only be posted to the participant once the research team member has spoken to the male partner and they have consented to participate in the study. Written consent forms will be returned by reply-paid post with the questionnaire and genital samples.

If the male partner declines to participate in the study and the female partner has already enrolled her data will not be used and her samples will be destroyed and she will be withdrawn from the study.

Follow up: All participants will be asked to collect samples at home the day following completion of the antibiotic (day 8), day 14, day 21 and at day 28.

For female participants this will involve self-collection of a vaginal swab and smearing a glass slide, and returning the swab, slide and questionnaire by post to investigators. Women will be taught how to self-collect samples at their enrolment visit and be given self-sampling instructions. Self-collected swabs for BV and PCR are equivalent to clinician-collected samples and these methods have been used extensively by investigators in previous studies[2,11,12,15,29-34].Table 1 outlines the study schedule. Questionnaires will assess in detail sexual practices over the prior week (including frequency and types of sex, condom use, change in partnerships etc), menstrual dates, vaginal symptoms and treatments, and adherence and side effects.

Reminder emails, SMS messages or phone calls will be used to prompt home specimen collection.

Male participants will be asked to self–collect penile skin swabs and a urine swab, and complete a questionnaire assessing in detail sexual practices over the prior week (including frequency and types of sex, condom use, change in partnerships etc), genital symptoms, and treatments, and adherence and side effects.

Reminder emails, SMS messages or phone calls will be used to prompt home specimen collection. Male and female participants will receive $50 each on completion of the study. Any women experiencing BV recurrence within the month will be offered a review appointment in clinic and recommended treatment for BV.

Table 1: Study timeline and sample collection summary for females

	**Clinic**	**Home**	**Home**	**Home**	**Home**
Study stage	D0	D8[a]	D14	D21	D28
Questionnaire	X	X	X	X	X
Examination	X				
Vaginal swabs (n)	2	1	1	1	1
Amsel's method	X				
Nugent's method	X	X	X	X	X
qPCR samples	X	X	X	X	X

D=day, n=number, [a]Post-antibiotic

Table 2: Study timeline and sample collection summary for males

	**Home/Clinic**[a]	**Home**	**Home**	**Home**	**Home**
Study stage	D0	D8[b]	D14	D21	D28
Questionnaire	X	X	X	X	X
Examination	X				
Penile skin swab (n)	1	1	1	1	1
Urine swab (n)	1	1	1	1	1
qPCR samples	1	1	1	1	1

D=day, n=number, [a]Clinic visit preferred if able to attend alternatively phone consultation at home, [b]post-antibiotic

Molecular testing of male and female samples Pre- antibiotic, day 8, 14, 21 and 28 samples will be tested by both quantitative PCR (qPCR) and shotgun metangenomic Illumina sequencing to investigate changes in the loads and abundance of key bacterial species in men and women following dual partner treatment. qPCR will be utilized as an efficient, unbiased and accurate approach for detecting, quantifying and monitoring changes in key bacterial spp. on the penile skin and urethra of males and in the vaginal microbiota in women. Bacterial targets have been selected based on our past studies[29,31,37] and our recent work using 16S rRNA gene pyrosequencing approaches[38[31,32,35,36]. Samples will also be stored to investigate the influence of male partner treatment on abundance & composition of bacterial communities in men and women using shotgun metagenomic Illumina sequencing. In brief genital and urine swabs will be batch processed using our established methods[31]. Extracted DNA will be subject to 13 qPCR assays for 8 key BVAB: *G.vaginalis*, *A.vaginae*, BVAB1, BVAB2, BVAB3, Megasphaera phylotype 1, *Prevotella* spp., *Leptotrichia & Sneathia* spp. (combined assay), as well as 4 *lactobacillus* spp, (*L.crispatus, L.iners, Ljensenii* and *L.gasseri* and the human beta-globin gene as a marker of sample adequacy, to detect inhibitors, and to normalise quantitation data[37]. Each bacterium-specific qPCR assay has been optimised to have an analytical sensitivity of 1-10

copies per reaction, and all targets have been designed to only detect the intended organism[31,36].

Blinding

This is an open label pilot study to determine the feasibility, acceptability and compliance of male partner treatment and to establish efficacy of combined antibiotic therapy against male penile skin and urethral BVAB. The data will be used to inform a large multicentre randomized controlled trial of male partner treatment to determine if it reduces BV recurrence in women.

Sample Size and Study Duration

50 couples will be enrolled as a convenience sample to determine the acceptability and feasibility of male partner treatment. They will be followed up weekly for a total of 4 weeks following enrolment. This will include women who are prescribed either of two first-line regimens of treatment: 1) oral metronidazole or 2) vaginal clindamycin.

Analysis S*econdary analyses* will be undertaken to examine the influence of male partner treatment on BVAB on the penile skin and urethra at one week and to examine the influence of dual partner treatment on BVAB in the male and female genital tract over one month. For this analysis, mean differences in the *organism loads* of BVAB between pre-treatment (Day O) and post-antibiotic (Day 8) samples will be compared in males using paired t-test or non-parametric equivalent. Mean differences in the *organism loads* of BVAB between endpoint (Day 28) and pre-treatment (Day O) samples will also be compared in males and females using paired t-test or non-parametric equivalent. These data will be used to inform sample size calcuations for a RCT of male partner treatment.

Feasibility of this RCT

A number of factors ensure high feasibility of this RCT. Our team has an extensive track record in BV research having conducted an RCT and 4 large observational cohort studies comprising of 2400 women and using the recruitment, sampling and longitudinal methods described in this application [2,11,12,15,33,34,38]. We have recruited 450 women with BV for an RCT exclusively at MSHC over 30 months[12,15], 1115 women from FPV and general practices over 15 months[2], and 450 women from the community for an incidence study over 18 months[33]. The follow-up for the 5 cohort studies ranged from 6 to 24 months, with assessment ranging from monthly to 3 monthly, and all achieved high retention rates (80-94%). Participants will be recruited from MSHC, which diagnoses over 550 cases of symptomatic BV (excluding repeat presentations) per annum. Data from a survey at our service indicates that this is an acceptable intervention to women with BV and their male partners. Of 123 women with BV handed an anonymous survey post-consultation to complete in the waiting room in 2014; 62 (50%) had a MSP and 27 (44%) indicated they would be willing to participate in a male partner treatment trial to determine if this reduced BV recurrence. Based on these data obtained from clinical studies at our service, we conservatively estimate that we will recruit 50 couples over 12 months and we will further enhance recruitment through advertising in FPV and high case load general practices.

Significance and Expected Outcomes

BV is a disease of high global burden in women and new effective therapies are a priority to improve cure rates, reduce sequelae and to assist with prevention strategies.

Epidemiological and microbiological evidence strongly supports sexual transmission of BV with reinfection from untreated partners likely to be the key driver of BV recurrence in women. No advances have been made in the treatment of BV in over 20 years and recurrence rates remain high. This is a pilot study in to determine the acceptability and feasibility of male partner treatment and to obtain microbiological data to show the impact of male partner treatment on BVAB in men and women. These data will be used to inform the design of a sufficiently powered placebo-controlled RCT to determine if male partner treatment, in addition to recommended antibiotic therapy for female cases reduces BV recurrence over six months. If shown to be effective, this intervention is uniquely positioned to be rapidly implemented in clinical practice and has the capacity to impact on the burden of BV in women in the community and thus reduce adverse sequelae.

References

1. Allsworth JE, Peipert JF. Prevalence of bacterial vaginosis: 2001-2004 national health and nutrition examination survey data. *Obstet Gynecol* 2007; 109: 114-120.
2. Bradshaw CS, Walker J, Fairley CK, et al. Prevalent and incident bacterial vaginosis are associated with sexual and contraceptive behaviours in young Australian women. *PLoS One* 2013; 8: e57688.
3. Chico RM, Mayaud P, Ariti C, et al. Prevalence of malaria and sexually transmitted and reproductive tract infections in pregnancy in sub-Saharan Africa: a systematic review. *Jama* 2012; 307: 2079-2086.
4. Myer L, Kuhn L, Stein ZA, et al. Intravaginal practices, bacterial vaginosis, and women's susceptibility to HIV infection: epidemiological evidence and biological mechanisms. *Lancet Infect Dis* 2005; 5: 786-794.
5. Cohen CR, Lingappa JR, Baeten JM, et al. Bacterial Vaginosis Associated with Increased Risk of Female-to-Male HIV-1 Transmission: A Prospective Cohort Analysis among African Couples. *PLoS Med* 2012; 9: e1001251.
6. Koumans EH, Markowitz LE, Berman SM, St Louis ME. A public health approach to adverse outcomes of pregnancy associated with bacterial vaginosis. *Int J Gynaecol Obstet* 1999; 67: S29-33.
7. Bilardi JE, Walker S, Temple-Smith M, et al. The burden of bacterial vaginosis: women's experience of the physical, emotional, sexual and social impact of living with recurrent bacterial vaginosis. *PLoS One* 2013; 8: e74378.
8. Fredricks DN, Fiedler TL, Thomas KK, et al. Targeted PCR for detection of vaginal bacteria associated with bacterial vaginosis. *J Clin Microbiol* 2007; 45: 3270-3276.
9. Swidsinski A, Mendling W, Loening-Baucke V, et al. An adherent Gardnerella vaginalis biofilm persists on the vaginal epithelium after standard therapy with oral metronidazole. *Am J Obstet Gynecol* 2008; 198: 97 e91-96.
10. Oduyebo OO, Anorlu RI, Ogunsola FT. The effects of antimicrobial therapy on bacterial vaginosis in non-pregnant women. *Cochrane Database Syst Rev* 2009: CD006055.
11. Bradshaw CS, Morton AN, Hocking J, et al. High recurrence rates of bacterial vaginosis over the course of 12 months after oral metronidazole therapy and factors associated with recurrence. *J Infect Dis* 2006; 193: 1478-1489.
12. Bradshaw CS, Pirotta M, De Guingand D, et al. Efficacy of oral metronidazole with vaginal clindamycin or vaginal probiotic for bacterial vaginosis: randomised placebo-controlled double-blind trial. *PLoS One* 2012; 7: e34540.
13. Potter J. Should sexual partners of women with bacterial vaginosis receive treatment? *Br J Gen Pract* 1999; 49: 913-918.
14. Mehta SD. Systematic Review of Randomized Trials of Treatment of Male Sexual Partners for Improved Bacterial Vaginosis Outcomes in Women. *Sex Transm Dis* 2012; 39: 822-830.
15. Bradshaw CS, Vodstrcil LA, Hocking JS, et al. Recurrence of Bacterial Vaginosis Is Significantly Associated With Posttreatment Sexual Activities and Hormonal Contraceptive Use. *Clin Infect Dis* 2013; 56: 777-786.
16. Fethers KA, Fairley CK, Hocking JS, et al. Sexual risk factors and bacterial vaginosis: a systematic review and meta-analysis. *Clin Infect Dis* 2008; 47: 1426-1435.

17. Sanchez S, Garcia PJ, Thomas KK, et al. Intravaginal metronidazole gel versus metronidazole plus nystatin ovules for bacterial vaginosis: a randomized controlled trial. *Am J Obstet Gynecol* 2004; 191: 1898-1906.
18. Schwebke JR, Desmond RA. A randomized trial of the duration of therapy with metronidazole plus or minus azithromycin for treatment of symptomatic bacterial vaginosis. *Clin Infect Dis* 2007; 44: 213-219.
19. Schwebke JR, Muzny CA, Josey WE. Role of Gardnerella vaginalis in the pathogenesis of bacterial vaginosis: a conceptual model. *J Infect Dis* 2014; 210: 338-343.
20. Bradshaw CS, Tabrizi SN, Read TRH, et al. Etiologies of non-gonococcal urethritis: bacteria, viruses and the association with oro-genital exposure. *J Infect Dis* 2006; 193: 336-345.
21. Piot P. Distribution of eight serotypes of Ureaplasma urealyticum in cases of non-gonococcal urethritis and of gonorrhoea, and in healthy persons. *Br J Vener Dis* 1976; 52: 266-268.
22. Schwebke JR, Rivers C, Lee J. Prevalence of Gardnerella vaginalis in male sexual partners of women with and without bacterial vaginosis. *Sex Transm Dis* 2009; 36: 92-94.
23. Nelson DE, Dong Q, Van Der Pol B, et al. Bacterial communities of the coronal sulcus and distal urethra of adolescent males. *PLoS One* 2012; 7: e36298.
24. Price LB, Liu CM, Johnson KE, et al. The effects of circumcision on the penis microbiome. *PLoS One* 2010; 5: e8422.
25. Gray RH, Wawer MJ, Serwadda D, Kigozi G. The role of male circumcision in the prevention of human papillomavirus and HIV infection. *J Infect Dis* 2009; 199: 1-3.
26. Swidsinski A, Loening-Baucke V, Mendling W, et al. Infection through structured polymicrobial Gardnerella biofilms (StPM-GB). *Histol Histopathol* 2014; 29: 567-587.
27. Amsel R, Totten PA, Spiegel CA, et al. Nonspecific vaginitis. Diagnostic criteria and microbial and epidemiologic associations. *Am J Med* 1983; 74: 14-22.
28. Nugent RP, Krohn MA, Hillier SL. Reliability of diagnosing bacterial vaginosis is improved by a standardized method of gram stain interpretation. *J Clin Microbiol* 1991; 29: 297-301.
29. Bradshaw CS, Morton A, Garland SM, et al. Higher-risk behavioral practices associated with bacterial vaginosis compared with vaginal candidiasis. *Obstet Gynecol* 2005; 106: 105-114.
30. Bradshaw CS, Morton AN, Garland SM, et al. An evaluation of a point-of-care test, BVBlue, and clinical and laboratory criteria for the diagnosis of bacterial vaginosis (BV). *J Clin Microbiol* 2005; 43: 1304-1308.
31. Bradshaw CS, Tabrizi SN, Fairley CK, et al. The association of Atopobium vaginae and Gardnerella vaginalis with bacterial vaginosis and recurrence after oral metronidazole therapy. *J Infect Dis* 2006; 194: 828-836.
32. Fethers K, Twin J, Fairley CK, et al. Bacterial vaginosis (BV) candidate bacteria: associations with BV and behavioural practices in sexually-experienced and inexperienced women. *PLoS One* 2012; 7: e30633.
33. Fethers KA, Fairley CK, Morton A, et al. Low Incidence of Bacterial Vaginosis in Cohort of Young Australian Women. *Sex Transm Dis* 2011; 38: 124-126.
34. Fethers KA, Fairley CK, Morton A, et al. Early sexual experiences and risk factors for bacterial vaginosis. *J Infect Dis* 2009; 200: 1662-1670.

35. Twin J, Bradshaw CS, Garland SM, et al. The potential of metatranscriptomics for identifying screening targets for bacterial vaginosis. *PLoS One* 2013; 8: e76892.
36. Tabrizi SN, Fairley CK, Bradshaw CS, Garland SM. Prevalence of Gardnerella vaginalis and Atopobium vaginae in virginal women. *Sex Transm Dis* 2006; 33: 663-665.
37. Tabrizi SN, Chen S, Cohenford MA, et al. Evaluation of real-time polymerase chain reaction assays for confirmation of Neisseria gonorrhoeae in clinical samples tested positive in the Roche COBAS AMPLICOR assay. *Sex Transm Dis* 2004; 80: 68-71.
38. Bradshaw CS, Walker SM, Vodstrcil LA, et al. The influence of behaviours and relationships on the vaginal microbiota of women and their female partners: The WOW Health Study. *J Infect Dis* 2013; Dec 29. [Epub ahead of print].
