# Supplementary material for: Combined oral and topical antimicrobial therapy for male partners of women with bacterial vaginosis: Acceptability, tolerability and impact on the genital microbiota of couples - A pilot study
Source: PLoS One. 2018 Jan 2;13(1):e0190199. doi: 10.1371/journal.pone.0190199 (PMC5749747; doi:10.1371/journal.pone.0190199)
Supplement: S1 Table — (DOCX) [file pone.0190199.s004.docx]

**Supplementary Table 1.** Negative controls included in the microbiota analysis

| **Control ID Number** | **DNA Extraction Control** | **Extraction method** | **Details of control ^a^** |
| --- | --- | --- | --- |
| 1, 2, 8 | PBS | Phenol / chloroform | Controls for phenol/chloroform extraction method |
| 3, 4, 5, 6 | PBS | MagnaPure | Controls for MagnaPure extraction method |
| 7 | Molecular grade water (Ambion) | Phenol / chloroform |  |
| 9 | MilliQ water | Phenol / chloroform |  |
|  | **PCR Reagent controls** |  |  |
| 10, 12, 14, 18 | Irradiated molecular grade water |  | Irradiated water was used for PCR setup, used as PCR reagent control |
| 11, 13, 15 | Molecular grade water (Ambion) |  | Water used to dilute out primers |
| 16, 17 | TE |  | TE used for resuspension of DNA from phenol-choloroform extraction |

Abbreviations: PBS, phosphate buffered saline solution; TE, Tris-EDTA buffer solution

^a^ Extraction negative controls were extracted alongside study samples.
