# Supplementary material for: Combined oral and topical antimicrobial therapy for male partners of women with bacterial vaginosis: Acceptability, tolerability and impact on the genital microbiota of couples - A pilot study
Source: PLoS One. 2018 Jan 2;13(1):e0190199. doi: 10.1371/journal.pone.0190199 (PMC5749747; doi:10.1371/journal.pone.0190199)
Supplement: S2 Table — (DOCX) [file pone.0190199.s005.docx]

**Supplementary Table 2.** Contaminants, identified based on criteria described in the methods, removed from OTU table prior to analysis

| **Taxon** | **No. of reads excluded from study specimens** |
| --- | --- |
| *Halomonas* | 326,390 |
| *Shewanella* | 104,939 |
| *Alcaligenes* | 13,501 |
| *Achromobacter* | 2,830 |
| *Variovorax* | 2,388 |
| *Ochrobactrum* | 2,026 |
| *Rhizobium* | 1,539 |
| *Bradyrhizobium* | 285 |
| *Methylobacterium* | 200 |
| *Rhizobiales*_1174-901-12 | 174 |
| *Aureimonas* | 98 |
| *Nitrobacter* | 90 |
| *Meiothermus* | 81 |
| *Bradyrhizobiaceae*_other | 54 |
| *Delftia* | 36 |
| *Beijerinckiaceae*_other | 31 |
| *Rhodopseudomonas* | 22 |
| *Cloacibacterium* | 20 |
| *Peredibacter* | 17 |
| *Thermus* | 15 |
| *Bosea* | 14 |
| *Rhizobiaceae*_Other | 10 |
| *Arthrobacter* | 9 |
| *Devosia* | 8 |
| *Rhizobiales*_other | 8 |
| *Aquabacterium* | 7 |
| *Xanthobacteraceae*_other | 4 |
