# Supplementary material for: Combined oral and topical antimicrobial therapy for male partners of women with bacterial vaginosis: Acceptability, tolerability and impact on the genital microbiota of couples - A pilot study
Source: PLoS One. 2018 Jan 2;13(1):e0190199. doi: 10.1371/journal.pone.0190199 (PMC5749747; doi:10.1371/journal.pone.0190199)
Supplement: S3 Table — (DOCX) [file pone.0190199.s006.docx]

**Supplementary Table 3.** Proportional abundances of the 30 most abundant taxa in vaginal specimens and penile skin specimens over the study period

|  | ***FEMALES* % abundance**  **mean, median**  **(IQR, Range)** | | | | | | |
| --- | --- | --- | --- | --- | --- | --- | --- |
| **Bacterial taxa** | **Baseline**  **(Day 0, N=17)^a^** | **Post antibiotic (Day 8, N=17)^a^** | **Study endpoint**  **(Day 28, N=16)^a^** | **p-value**  **Day 0-8^b^** | **q-value**  **Day 0-8^c^** | **p-value**  **Day 0-28^b^** | **q-value**  **Day 0-28^c^** |
| ***Gardnerella*** | 33.8, 31.7  (25.7-40.6, 0-79.1) | 9.9, 0  (0-0.1, 0-99.2) | 4.1, 0.1  (0.1-1.9, 0-44.7) | **0.007** | **0.030** | **0.001** | **0.012** |
| ***Prevotella*** | 14.2, 15.7  (7.1-21.9, 0-28.6) | 0.6, 0  (0-0, 0-9.5) | 3.4, 0  (0-0.7, 0-31.8) | **<0.001** | **0.012** | **0.001** | **0.012** |
| ***Sneathia*** | 13.0, 5.5  (0.6-22.2, 0-52.7) | 0.1, 0  (0-0, 0-0.8) | ND | **<0.001** | **0.012** | **0.001** | **0.012** |
| ***Lactobacillus iners*** | 11.8, 1.9  (0.1-7.4, 0-89.5) | 61.4, 97.5  (7.9-99.8, 0-100) | 62.7, 91.6  (2.6-97.9, 0-99.8) | **0.008** | **0.033** | **0.005** | **0.024** |
| ***Megasphaera*** | 4.2, 1.8  (0-5.2, 0-16.4) | 0.04, 0  (0-0, 0-0.7) | ND | **0.001** | **0.012** | **0.002** | **0.014** |
| ***Dialister*** | 3.6, 2.1  (0.6-4.4, 0-17.2) | 0.2, 0  (0-0.1, 0-2.5) | 0.7, 0  (0-0.1, 0-8.6) | **<0.001** | **0.012** | **0.016** | 0.056 |
| ***Atopobium*** | 3.6, 3.0  (1-5.4, 0-12.9) | 0.01, 0  (0-0, 0-0.1) | 0.03, 0  (0-0, 0-0.6) | **<0.001** | **0.012** | **<0.001** | **0.012** |
| ***Enterorhabdus*** | 2.3, 1.7  (1.3-3.2, 0-8.1) | ND | 0.2, 0  (0-0, 0-2.6) | **<0.001** | **0.012** | **0.002** | **0.013** |
| ***Fastidiosipila*** | 1.9, 1.2  (0-3.6, 0-5.8) | 0.1, 0  (0-0, 0-1.6) | ND | **0.002** | **0.014** | **0.003** | **0.018** |
| ***Shuttleworthia*** | 1.7, 0  (0-0, 0-29.2) | ND | ND | 0.317 | 0.449 | - | - |
| ***Fusobacterium*** | 1.5, 0  (0-0, 0-16.4) | ND | ND | **0.046** | 0.118 | **0.046** | 0.118 |
| ***Anaerococcus*** | 1.2, 0.3  (0-0.6, 0-6.5) | 0, 0  (0-0, 0-0) | 0.2, 0  (0-0.2, 0-1.7) | **<0.001** | **0.012** | **0.017** | 0.057 |
| ***Finegoldia*** | 0.8, 0.1  (0-0.1, 0-11.4) | 0.1, 0  (0-0, 0-2.5) | 0.3, 0  (0-0.1, 0-2.6) | **0.003** | **0.018** | 0.207 | 0.354 |
| ***Peptostreptococcus*** | 0.8, 0  (0-0.3, 0-10.1) | ND | 0.1, 0  (0-0, 0-1.5) | **0.009** | **0.036** | **0.009** | **0.036** |
| ***Veillonella*** | 0.8, 0  (0-0.1, 0-9.0) | ND | 0.02, 0  (0-0, 0-0.3) | **0.026** | 0.079 | 0.227 | 0.383 |
| ***Candidate division TM7*** | 0.8, 0  (0-0, 0-12.8) | 0.1, 0  (0-0, 0-1.4) | ND | 0.317 | 0.449 | 0.317 | 0.449 |
| ***Lactobacillus crispatus*** | 0.6, 0  (0-0, 0-7.7) | 5.9, 0  (0-0, 0-99.4) | 5.6, 0  (0-0, 0-83.3) | 1 | 1.000 | 0.945 | 0.953 |
| ***Parvimonas*** | 0.6, 0.5  (0-0.8, 0-2.6) | ND | ND | **0.001** | **0.012** | **0.002** | **0.014** |
| ***Gemella*** | 0.5, 0  (0-0.3, 0-6.3) | ND | 0.01, 0  (0-0, 0-0.1) | **0.005** | **0.025** | **0.014** | 0.054 |
| ***Porphyromonas*** | 0.4, 0  (0-0.3, 0-4.0) | ND | 0.01, 0  (0-0, 0-0.1) | **0.015** | 0.054 | **0.015** | 0.054 |
| ***Streptococcus*** | 0.3, 0  (0-0, 0-4.7) | 0.7, 0  (0-0, 0-8.1) | 1.7, 0  (0-0.3, 0-23.3) | 0.614 | 0.713 | 0.384 | 0.521 |
| ***Aerococcus*** | 0.3, 0.2  (0-0.4, 0-1.6) | 0.2, 0  (0-0.1, 0-1.3) | 0.8, 0  (0-0.1, 0-9.6) | 0.613 | 0.713 | 0.250 | 0.409 |
| ***Lactobacillus fornicalis*** | 0.1, 0  (0-0, 0-0.6) | 0.04, 0  (0-0, 0-0.5) | 4.4, 0  (0-0, 0-39.4) | 0.635 | 0.713 | 0.546 | 0.678 |
| ***Corynebacterium*** | 0.02, 0  (0-0, 0-0.2) | 1.0, 0  (0-0, 0-16.6) | 0.5, 0.1  (0-0.2, 0-5.9) | 0.288 | 0.447 | **0.048** | 0.120 |
| ***Enterobacter*** | ND | 8.4, 0  (0-0, 0-87.7) | 0.2, 0  (0-0, 0-3.5) | 0.084 | 0.168 | 0.158 | 0.277 |
| ***Kluyvera*** | ND | 0.7, 0  (0-0, 0-11.8) | ND | 0.317 | 0.449 | - | - |
| ***Escherichia/Shigella*^d^** | ND | 5.6, 0  (0-0, 0-86.6) | 11.0, 0  (0-0, 0-88.7) | **0.046** | 0.118 | 0.084 | 0.168 |
| **Bacillales other** | ND | 2.7, 0  (0-0, 0-43.7) | 0.4, 0  (0-0, 0-4.3) | 0.158 | 0.277 | 0.084 | 0.168 |
| ***Staphylococcus*** | ND | 1.2, 0  (0-0, 0-19.4) | 1.4, 0  (0-0.1, 0-20.8) | 0.084 | 0.168 | 0.046 | 0.118 |
| ***Lactobacillus gasseri*** | ND | 0.02, 0  (0-0, 0-0.3) | 0.6, 0  (0-0, 0-9.0) | 0.158 | 0.277 | 0.084 | 0.168 |
|  | ***MALES (PENILE SKIN)* % abundance**  **mean, median**  **(IQR, Range)** | | | | | | |
| **Bacterial taxa** | **Baseline**  **(Day 0, N=16)^e^** | **Post antibiotic**  **(Day 8, N=16) ^e,f^** | **Study endpoint (Day 28, N=15)^e^** | **p-value**  **Day 0-8^b^** | **q-value**  **Day 0-8^c^** | **p-value**  **Day 0-28^b^** | **q-value**  **Day 0-28^c^** |
| ***Finegoldia*** | 24.6, 24.9  (3.0-44.6,0-58.7) | 2.2, 0  (0-0,0-34.5) | 14.8, 9.2  (2.9-21.8,0-58.6) | **0.002** | **0.013** | 0.057 | 0.137 |
| ***Corynebacterium*** | 17.3, 12.6  (5.4-27.4,1.3-50.3) | 21.1, 13.9  (1.9-31.5,0.5-70.0) | 29.4, 22.6  (13.3-41.4,1.2-88.7) | 0.679 | 0.738 | 0.156 | 0.277 |
| ***Peptoniphilus*** | 7.3, 5.4  (0.7-12.8,0-22.2) | 1.2, 0  (0-0,0-18.0) | 5.6, 3.6  (0-4.9,0-25.2) | **0.001** | **0.012** | 0.244 | 0.406 |
| ***Prevotella*** | 7.2, 1.8  (0.1-9.6,0-42.9) | 0.7, 0  (0-0.1,0-9.8) | 7.8, 0.3  (0-12.6,0-34.6) | **0.001** | **0.012** | 0.320 | 0.449 |
| ***Staphylococcus*** | 5.0, 1.0  (0.1-5.0,0-27.6) | 32.5, 14.8  (0.4-72.5,0-98.5) | 11.8, 0.8  (0.1-12.1,0-75.9) | **0.049** | 0.121 | 0.394 | 0.528 |
| ***Anaerococcus*** | 4.0, 3.7  (1.1-7.0,0-9.6) | 0.6, 0  (0-0,0-8.6) | 4.5, 1.4  (0.5-9.6,0-20.7) | **0.004** | **0.019** | 0.650 | 0.716 |
| ***Streptococcus*** | 3.9, 0.1  (0-0.8,0-35.0) | 0.2, 0  (0-0.2,0-0.9) | 2.1, 0.1  (0-0.9,0-13.3) | **0.035** | 0.101 | 0.909 | 0.925 |
| ***Escherichia/Shigella*^d^** | 3.7, 0  (0-0,0-58.5) | 10.0, 0  (0-0.9,0-57.7) | 0.1, 0  (0-0,0-1.27) | 0.302 | 0.449 | 0.564 | 0.688 |
| ***Dialister*** | 2.6, 0.1  (0-4.1,0-13.8) | 0.1, 0  (0-0,0-1.7) | 1.5, 0.2  (0-2.6,0-5.6) | **0.003** | **0.018** | 0.079 | 0.168 |
| ***Actinomyces*** | 2.5, 0.8  (0-1.6,0-15.3) | 0.31, 0  (0-0.05,0-2.45) | 1.5, 0.6  (0-0.9,0-16.1) | **0.017** | 0.057 | 0.565 | 0.688 |
| ***Gardnerella*** | 2.1, 0  (0-0.1,0-22.7) | 1.6, 0  (0-0.1,0-21.6) | 0.1, 0  (0-0.1,0-1.0) | 0.447 | 0.573 | 0.872 | 0.910 |
| ***Neisseria*** | 2.0, 0  (0-0,0-26.1) | ND | 0.02, 0  (0-0,0-0.3) | 0.084 | 0.168 | 0.268 | 0.427 |
| ***Brevundimonas*** | 2.0, 0  (0-0.1,0-21.6) | 0.2, 0  (0-0,0-3.1) | 0.2, 0  (0-0,0-2.4) | **0.046** | 0.118 | 0.632 | 0.713 |
| ***Enterobacter*** | 1.8, 0  (0-0,0-28.4) | 3.1, 0  (0-0,0-35.9) | ND | 0.084 | 0.168 | 0.317 | 0.449 |
| ***Veillonella*** | 1.4, 0  (0-0.2,0-17.0) | 0.2, 0  (0-0,0-1.5) | 0.2, 0  (0-0.3,0-1.6) | 0.092 | 0.178 | 0.904 | 0.925 |
| ***Peptostreptococcus*** | 1.3, 0  (0-0.6,0-15.4) | ND | 0.4, 0  (0-0,0-3.6) | **0.026** | 0.079 | 0.348 | 0.483 |
| ***Micrococcus*** | 1.2, 0  (0-0.3,0-12.8) | 0.5, 0  (0-0,0-5.2) | 0.1, 0  (0-0,0-1.8) | 0.080 | 0.168 | **0.015** | 0.054 |
| ***Bacillales other*** | 0.9, 0  (0-0.3,0-11.5) | 1.6, 0  (0-0.9,0-15.9) | 0.7, 0  (0-0.6,0-6.9) | 0.881 | 0.912 | 0.575 | 0.692 |
| ***Zimmermannella*** | 0.7, 0  (0-0,0-10.9) | 0.01, 0  (0-0,0-0.2) | 0.01, 0  (0-0,0-0.1) | 0.514 | 0.645 | 0.510 | 0.645 |
| ***Corynebacteriaceae other*** | 0.7, 0  (0-0.1,0-10.3) | 0.1, 0  (0-0,0-0.6) | 0.1, 0  (0-0.1,0-1.2) | 0.645 | 0.716 | 0.768 | 0.820 |
| ***Haemophilus*** | 0.7, 0  (0-0,0-8.9) | 0.1, 0  (0-0,0-1.5) | 0.4, 0  (0-0,0-5.9) | 0.271 | 0.427 | 0.632 | 0.713 |
| ***Porphyromonas*** | 0.5, 0.1  (0-0.8,0-2.6) | 0.2, 0  (0-0,0-2.5) | 2.0, 0  (0-0.5,0-26.4) | 0.034 | 0.101 | 0.425 | 0.558 |
| ***Gemella*** | 0.4, 0  (0-0.1,0-4.5) | 0.4, 0  (0-0,0-7.1) | 0.2, 0  (0-0,0-2.6) | 0.371 | 0.510 | 0.400 | 0.530 |
| ***Rothia*** | 0.3, 0  (0-0,0-4.0) | 0.01, 0  (0-0,0-0.2) | 0.4, 0  (0-0,0-6.3) | 0.302 | 0.449 | 0.681 | 0.738 |
| ***Enhydrobacter*** | 0.2, 0  (0-0.1,0-2.1) | 0.1, 0  (0-0,0-1.4) | 10.2, 0  (0-0,0-92.7) | 0.204 | 0.353 | 0.811 | 0.854 |
| ***Campylobacter*** | 0.2, 0  (0-0.1,0-1.5) | ND | 0.5, 0  (0-0,0-7.4) | **0.026** | 0.079 | 0.437 | 0.567 |
| ***Lactobacillus iners*** | 0.1, 0  (0-0.1,0-0.6) | 6.0, 0  (0-0,0-92.7) | 3.0, 0  (0-0.2,0-31.8) | 0.616 | 0.713 | 0.771 | 0.820 |
| ***Enterobacteriaceae otu1*** | 0.1, 0  (0-0,0-0.6) | 2.2, 0  (0-0,0-33.6) | ND | 0.589 | 0.702 | 0.084 | 0.168 |
| ***Pseudomonas*** | 0.02, 0  (0-0,0-0.2) | 12.5, 0  (0-0.3,0-98.2) | ND | 0.087 | 0.172 | 0.157 | 0.277 |
| ***Stenotrophomonas*** | 0.01, 0  (0-0,0-0.1) | 0.9, 0  (0-0.1,0-11.1) | 0.1, 0  (0-0,0-1.0) | 0.144 | 0.273 | 0.268 | 0.427 |

Abbreviations: IQR, interquartile range; ND, not detected

^a^ Seventeen women provided vaginal specimens for day 0 and 8 paired comparisons, and 16 provided vaginal specimens for day 0 and 28 paired comparisons.

^b^ Bacterial abundances were compared between paired samples using the Wilcoxon paired test.

^c^ FDR corrected p-value as assessed by the Wilcoxon paired test

^d^ *Escherichia* and *Shigella* cannot be reliably distinguished by their 16S rRNA gene. As such, they are combined here as one taxon *Escherichia/Shigella*.

^e^ Sixteen males provided penile specimens for day 0 and 8 paired comparisons, and 15 provided penile specimens for day 0 and 28 paired comparisons.

^f^ Two day 8 penile specimens failed to meet the sequence depth threshold and were substituted with day 14 specimens
