# Supplementary material for: Combined oral and topical antimicrobial therapy for male partners of women with bacterial vaginosis: Acceptability, tolerability and impact on the genital microbiota of couples - A pilot study
Source: PLoS One. 2018 Jan 2;13(1):e0190199. doi: 10.1371/journal.pone.0190199 (PMC5749747; doi:10.1371/journal.pone.0190199)
Supplement: S4 Table — (DOCX) [file pone.0190199.s007.docx]

**Supplementary Table 4.** Correlation of specific bacterial taxa between vaginal and cutaneous penile specimens of couples at baseline, day 8 and day 28

| **Day** | **Bacterial taxon^a^** | **Rho^a^** | **q value^b^** |
| --- | --- | --- | --- |
| *Baseline (Day 0)^c^* | *Atopobium* | 0.470 | 0.20 |
|  | *Peptostreptococcus* | 0.459 | 0.20 |
|  | *Veillonella* | 0.398 | 0.30 |
|  | *Parvimonas* | 0.345 | 0.41 |
|  | *Gardnerella* | 0.332 | 0.41 |
|  | *Lactobacillus iners* | 0.325 | 0.41 |
|  | *Finegoldia* | 0.283 | 0.50 |
|  | *Gemella* | 0.273 | 0.50 |
|  | *Fastidiosipila* | 0.212 | 0.61 |
|  | *Sutterella* | 0.178 | 0.68 |
|  | *Megasphaera* | 0.184 | 0.68 |
|  | *Anaerococcus* | 0.123 | 0.80 |
|  | *Aerococcus* | 0.073 | 0.90 |
|  | *Sneathia* | 0.046 | 1.00 |
|  | *Enterorhabdus* | 0.015 | 1.00 |
|  | *Prevotella* | -0.002 | 1.00 |
|  | *Peptoniphilus* | -0.026 | 1.00 |
|  | *Porphyromonas* | -0.097 | 0.84 |
|  | *Dialister* | -0.143 | 0.75 |
| *Day 8^d^* | *Gardnerella* | 0.336 | 0.48 |
|  | *Dialister* | 0.273 | 0.56 |
|  | *Ureaplasma* | 0.193 | 0.68 |
|  | *Lactobacillus iners* | 0.118 | 0.84 |
| *Day 28^e^* | *Dialister* | 0.720 | 0.05 |
|  | *Prevotella* | 0.707 | 0.05 |
|  | *Peptoniphilus* | 0.558 | 0.20 |
|  | *Anaerococcus* | 0.545 | 0.20 |
|  | *Corynebacterium* | 0.541 | 0.20 |
|  | *Streptococcus* | 0.498 | 0.24 |
|  | *Aerococcus* | 0.442 | 0.33 |
|  | *Gardnerella* | 0.275 | 0.56 |
|  | *Lactobacillus iners* | 0.273 | 0.56 |
|  | *Finegoldia* | 0.029 | 0.98 |

^a^ Spearman Correlation Coefficient: rho 0-0.19 “very weak”; 0.2-0.39 “weak”; 0.4-0.59 “moderate”; 0.6-0.79 “strong”; 0.8-1 “very strong”

^b^ *P*-value false discovery rate adjustment for multiple comparisons was performed using the Benjamini-Hochberg procedure; a *q*-value <0.05 was deemed significant

^c^ Only those bacteria present in 30% or more of vaginal samples collected at baseline were included in the correlation analysis. Analysis included a total of 20 paired specimens (i.e. data included from 20 couples is in this analysis).

^d^ Only those bacteria present in 30% or more of vaginal samples collected at day 8 were included in the correlation analysis. Analysis included a total of 16 paired specimens (i.e. data included from 16 couples in this analysis)

^e^ Only those bacteria present in 30% or more of vaginal samples collected at day 28 were included in the correlation analysis. Analysis included a total of 15 paired specimens (i.e. data included from 15 couples in this analysis)
