# Supplementary figures and images for: Combined oral and topical antimicrobial therapy for male partners of women with bacterial vaginosis: Acceptability, tolerability and impact on the genital microbiota of couples - A pilot study
Source: PLoS One. 2018 Jan 2;13(1):e0190199. doi: 10.1371/journal.pone.0190199 (PMC5749747; doi:10.1371/journal.pone.0190199)

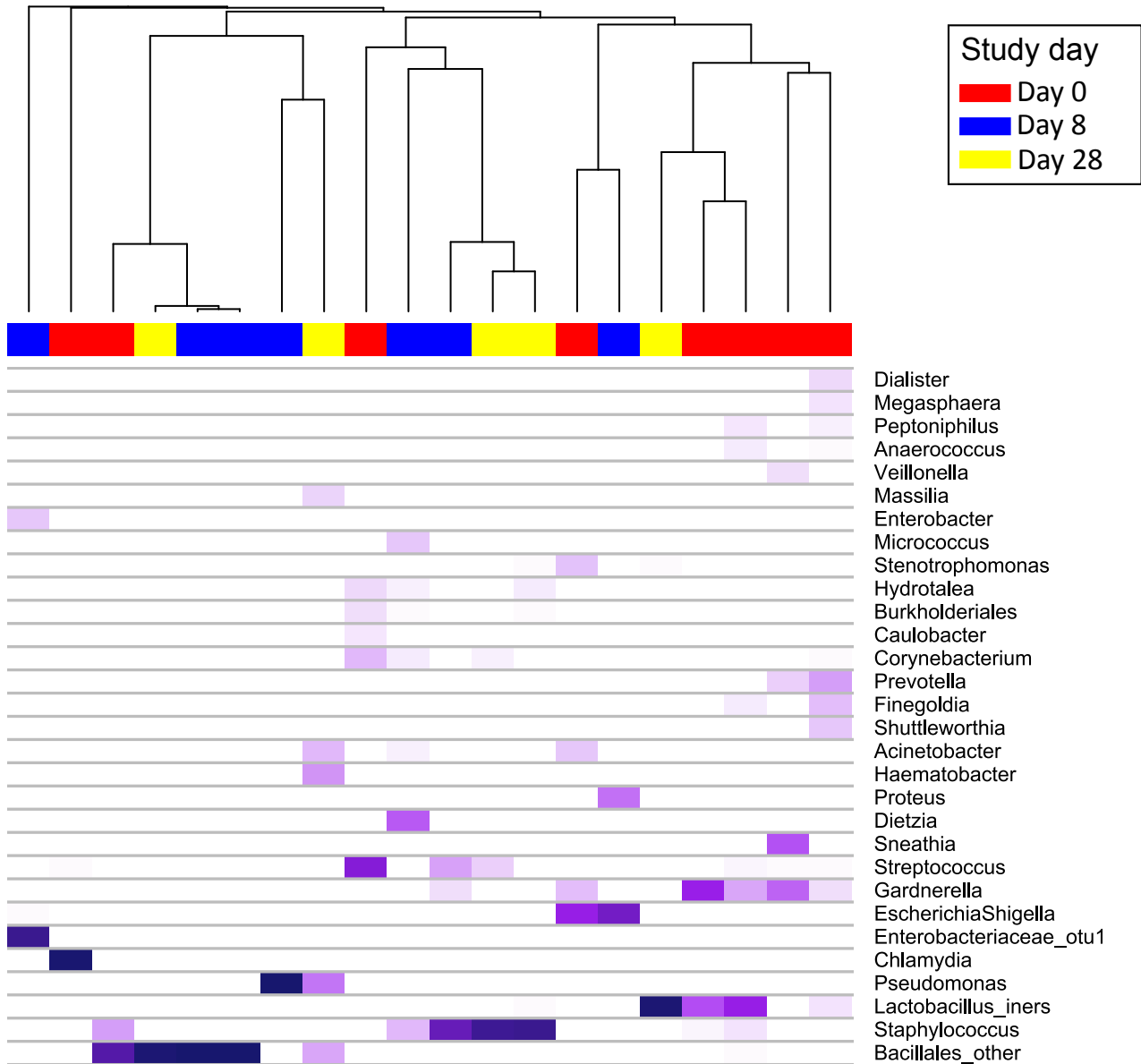

Supplement: S1 Fig — Each vertical line represents the bacterial composition of one urine specimen. Only the 30 most abundant taxa found in urine specimens are included in the heatmap. Study day is displayed above the heatmap in red (day 0), blue (day 8) and yellow (day 28). (PDF) [file pone.0190199.s008.pdf]

Similarity (Bray-Curtis)

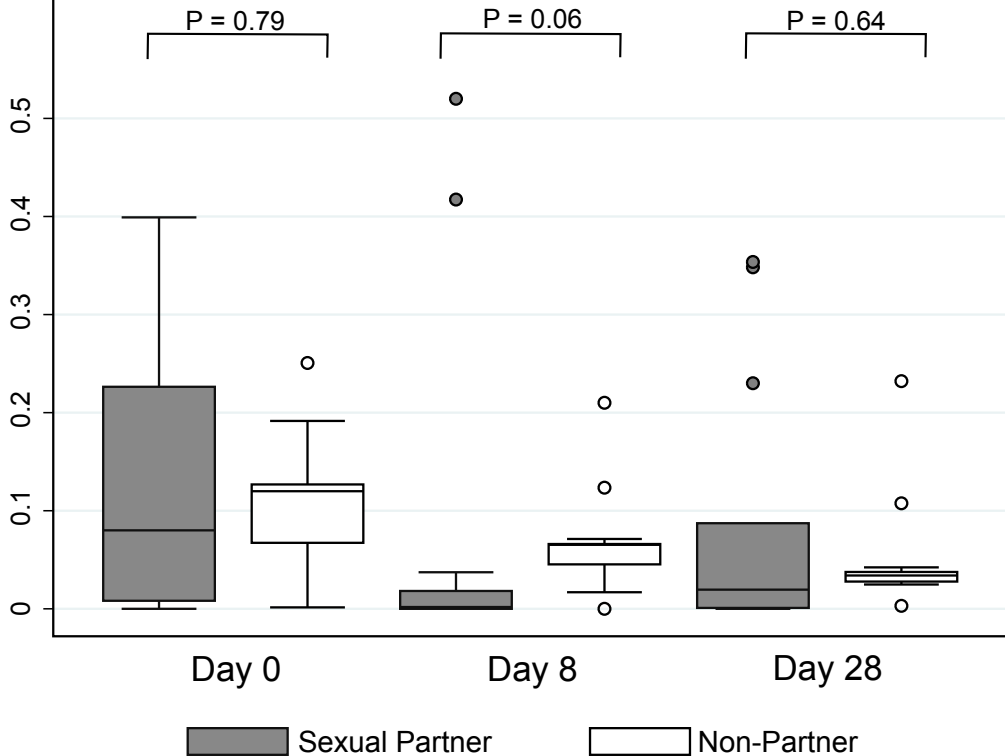

Supplement: S2 Fig — Box and whisker plots show median, Interquartile range (IQR), and the most extreme values within 1.5 IQR of the nearest quartile (dots show outliers). A lower Bray-Curtis similarity score indicates the vaginal and penile microbiota of couples is dissimilar. Comparisons are made between specimens from couples collected before treatment (D0), immediately after treatment at day 8 (D8) and three weeks after cessation of treatment at day 28 (D28). Differences in the similarity of bacterial communities of sexual partners compared to non-partners were assessed at each time point using the Wilcoxon signed-rank test. (PDF) [file pone.0190199.s009.pdf]
